# The causal relationship of female infertility and psychiatric disorders in the European population: a bidirectional two-sample Mendelian randomization study

**DOI:** 10.1186/s12905-024-02888-5

**Published:** 2024-01-19

**Authors:** Pengfei Zeng, Hanxiao Zhang, Liyue Lu, Yanting Li, Tong Yu, Jun Zhang, Hang Zhou

**Affiliations:** 1 School of Clinical Medicine, Chengdu University of Traditional Chinese Medicine, Chengdu, Sichuan China; 2 Faculty of Medicine, Université Paris-Saclay, Villejuif, France; 3 School of Shuguang Clinical Medicine, Shanghai University of Traditional Chinese Medicine, Shanghai, China; 4 School of Acu-Mox and Tuina, Chengdu University of Traditional Chinese Medicine, Chengdu, Sichuan China; 5 Department of Gynecology, Guangan Hospital of Traditional Chinese Medicine, Guangan, Sichuan China; 6 Department of Gynecology, Meishan Women and Children’s Hospital Alliance Hospital of West China Second University Hospital, Sichuan University, Meishan, Sichuan China; 7 School of Basic Medicine, Chengdu University of Traditional Chinese Medicine, Chengdu, Sichuan China

**Keywords:** Mendelian randomization, Causality, Female infertility, Psychiatric disorders

## Abstract

**Background:**

Infertility affects many couples globally, causing physical, emotional, and financial burdens. While observational studies suggest a link between psychiatric disorders and female infertility, causal relationships remain uncertain. Mendelian randomization analysis, using genome-wide association studies data, minimizes confounding factors and reverse causation, providing valuable insights into causal associations.

**Methods:**

We conducted Mendelian randomization analysis to explore the potential causal relationship between female infertility and psychiatric disorders. Genome-wide association studies summary data for female infertility (112,105 individuals of European ancestry, comprising 11,442 cases and 100,663 controls), depression (807,553 individuals of European ancestry, comprising 246,363 cases and 561,190 controls), anxiety (21,763 individuals of European ancestry, comprising 7,016 cases and 14,745 controls), bipolar disorder (51,710 individuals of European ancestry, comprising 20,352 cases and 31,358 controls), and eating disorders (72,517 individuals of European ancestry, comprising 16,992 cases and 55,525 controls) were utilized. Instrumental variables were selected based on significant single nucleotide polymorphisms associated with each phenotype. We assessed instrumental variable strength, examined confounding factors, and employed inverse variance weighting, weighted median, and MR-Egger approaches for analysis.

**Results:**

Our analysis included 85 single nucleotide polymorphisms for female infertility and 62 single nucleotide polymorphisms for psychiatric disorders. Results suggest a potential causal relationship between depression and female infertility, with both inverse variance weighting and weighted median methods showing increased infertility risk in depressed patients. Evidence is weak regarding bipolar disorder not increasing female infertility risk. We found no evidence supporting causal links between anxiety, eating disorders, and female infertility. Similarly, no causal relationship was found between female infertility and psychiatric disorders in the opposite direction. Sensitivity analyses and tests for heterogeneity and polymorphism supported result robustness.

**Conclusions:**

This analysis provides evidence for a potential causal relationship between depression and female infertility. Addressing depression in infertile women may improve fertility outcomes. Further research is needed to explore underlying mechanisms and potential interventions for improving fertility outcomes in women with psychiatric disorders.

**Supplementary Information:**

The online version contains supplementary material available at 10.1186/s12905-024-02888-5.

## Background

Infertility is a medical condition characterized by the failure to achieve a clinical pregnancy after 12 months of regular and unprotected sexual intercourse [1]. This condition affects approximately 8–12% of couples of reproductive age worldwide, with potentially higher incidence rates observed in developing countries [1]. Female factors contribute to 33–41% of infertility cases, while 9–39% of cases involve a combination of male and female factors [1]. Assisted reproductive technology (ART) has made significant technological advancements and has become the standard treatment for infertility in recent decades. However, the success rate of ART remains relatively modest, with clinical pregnancy rates (35%) only marginally surpassing those of natural conception (20%) [2, 3]. Furthermore, although ART is generally considered safe, research indicates a heightened risk of infection, ovarian hyperstimulation syndrome, multiple pregnancies, placentation disorders, and adverse pregnancy outcomes, including pregnancy-induced hypertension, gestational diabetes mellitus, placenta previa, placental abruption, and low birth weight [4]. Moreover, the overall costs associated with female infertility treatments are substantial, placing a significant economic burden on individuals, families, healthcare systems, and society at large. A study conducted in Israel revealed that 5.4% of total health maintenance organization expenditure was allocated to female infertility costs [5]. Female infertility patients often endure a substantial psychological burden due to the side effects of treatments, uncertainty about treatment efficacy, and the high financial implications. Furthermore, the prevalence of psychiatric disorders has been found to be higher in female infertility patients compared to the general population [6, 7].

Numerous observational studies have extensively investigated the association between psychiatric disorders and female infertility as a common complication [8, 9]. Previous investigations have consistently demonstrated an increased risk of depression [8], anxiety [10], and eating disorders [11]. in women with infertility. Conversely, psychiatric disorders such as depression, anxiety, bipolar disorder, and eating disorders can also influence female fertility [12–14]. Moreover, psychiatric disorders have been linked to clinical outcomes in ART treatments [15, 16]. However, the findings from these studies have been subject to controversy due to limitations, including a limited number of studies, methodological weaknesses, and significant heterogeneity across studies. Furthermore, these studies establish a correlation between mental illness and infertility but fail to establish a causal relationship due to the potential presence of confounding variables or reverse causation.

Mendelian randomization (MR) analysis, utilizing summary statistics from genome-wide association studies (GWAS), is a robust and efficient approach to establish causal associations between exposure phenotypes and outcome phenotypes. By mitigating the impact of confounding factors and reverse causation, MR analysis serves as a valuable tool for investigating causal relationships [17].

Considering the limited evidence regarding the link between female infertility and psychiatric disorders, we conducted a two-sample Mendelian randomization (MR) analysis to investigate the causal effects of female infertility on the risks of depression, anxiety, bipolar disorder, and eating disorders. Our study aims to unveil potential genetic mechanisms underlying the connection between female infertility and psychiatric disorders, offering scientific evidence for primary disease prevention.

## Methods

No further ethics approval was deemed necessary for this study as it entailed a reanalysis of previously gathered and published data. Hence, no novel data collection was conducted, and the utilization of publicly available data rendered it exempt from additional ethics approval.

### Study design

**Fig. 1:**
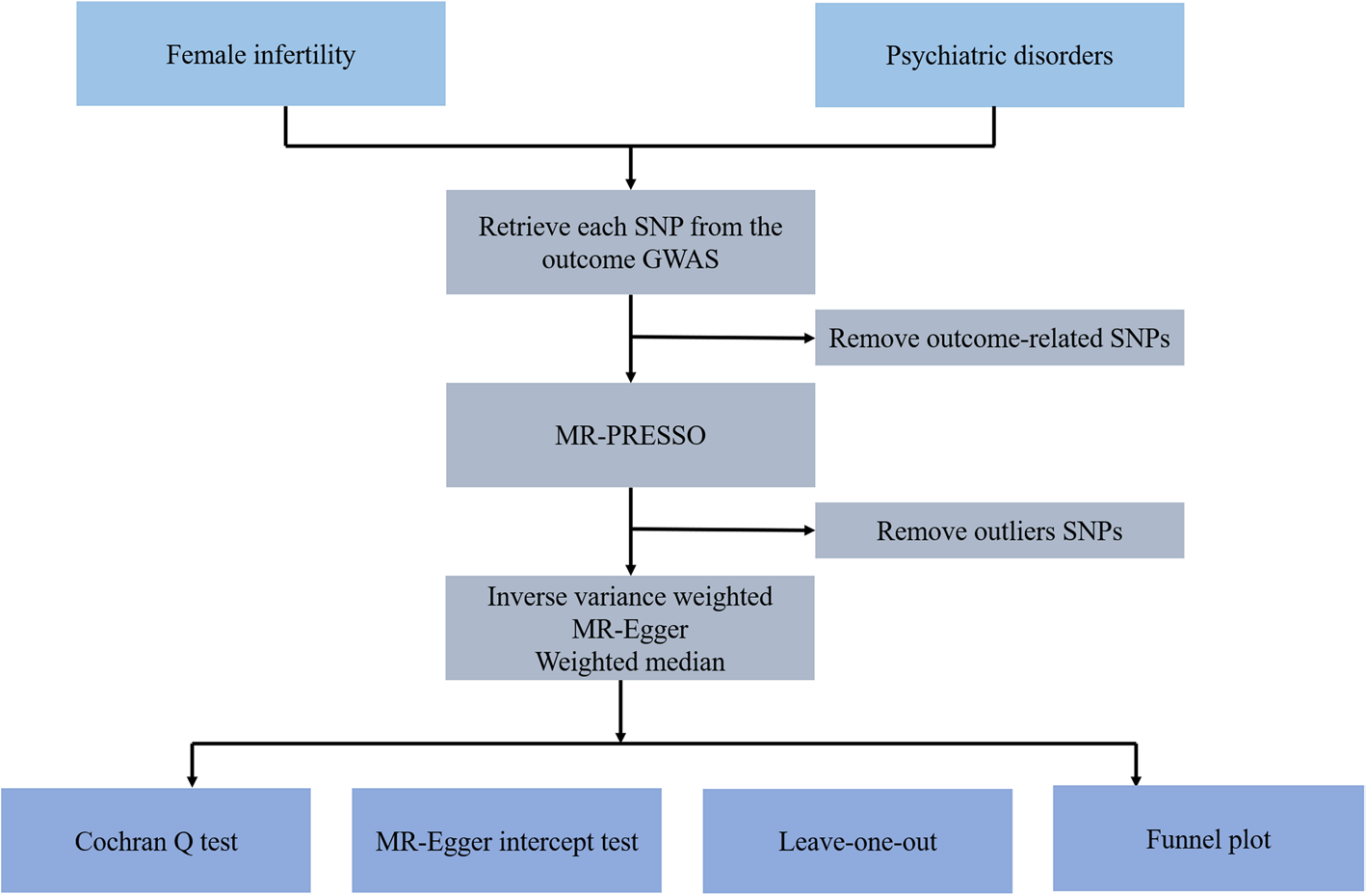
Flow chart of the MR study design

We employed bidirectional MR methodology to investigate the potential causal association between female infertility and psychiatric disorders, as depicted in Fig. 1. In order to accomplish this, we obtained relevant genetic variants from comprehensive GWAS conducted on female infertility, depression, anxiety, bipolar disorder, and eating disorders. We employed psychiatric disorders-related GWAS data (depression, anxiety, bipolar disorder, and eating disorders) as the exposure and female infertility GWAS data as the outcome, conducting the MR analysis to scrutinize their causal relationship. Subsequently, we conducted a reverse MR analysis, using female infertility GWAS data as the exposure and psychiatric disorders-related GWAS data (depression, anxiety, bipolar disorder, and eating disorders) as the outcome, to further examine the causal dynamics between these variables. To ensure the robustness and validity of our bidirectional MR analysis, we made several assumptions, including: (1) establishing that the single nucleotide polymorphisms (SNPs) derived from GWAS and employed as instrumental variables (IVs) exhibit an association with the exposures, (2) ensuring the genetic variants are independent of any potential confounding factors, and (3) verifying that the IVs solely influence the outcome through the exposure [18, 19].

### GWAS summary data for female infertility risk factors

We obtained data on female infertility from the FinnGen GWAS Summary Statistics R8 release (public release: 1 December 2022, https://r8.finngen.fi/, accessed on 21 April 2023) [20], which included information from 112,105 individuals of European ancestry, comprising 11,442 cases and 100,663 controls, Detailed information about exposure for female infertility is provided in Table 1.

**Table 1:** Summary of female infertility

Exposure	Ancestry	Data source	Number of cases	Number of controls	Year	Outcome	*F*
Female infertility	European	finngen_R8_N14_FEMALEINFERT	11,442	100,663	2022	Depression	24.30
						Anxiety	22.81
						Bipolar disorder	23.23
						Eating Disorders	24.43

### GWAS summary data for psychiatric disorders

We obtained data on psychiatric disorders from the Psychiatric Genomics Consortium (https://pgc.unc.edu/about-us/, accessed on 21 April 2023). Data pertaining to depression were acquired from a comprehensive genome-wide meta-analysis encompassing a total of 807,553 individuals of European ancestry (246,363 cases and 561,190 controls) [21]. The anxiety-related data were obtained from a meta-analysis specifically focused on anxiety, encompassing a cohort of 21,763 individuals of European ancestry (7,016 cases and 14,745 controls) [22]. Similarly, data concerning bipolar disorder were collected from a GWAS study that involved a cohort of 51,710 individuals of European ancestry (20,352 cases and 31,358 controls) [23]. Lastly, data concerning eating disorders were obtained from a GWAS meta-analysis comprising a cohort of 72,517 individuals of European ancestry (16,992 cases and 55,525 controls) [24]. Detailed information about exposure for psychiatric disorders is provided in Table 2.

**Table 2:** Summary of psychiatric disorders

Exposure	Ancestry	Data source	Number of Cases	Number of Controls	Year	Outcome	*F*
Depression	European	[21]	246,363	561,190	2019	Female infertility	45.79
Anxiety		[22]	7,016	14,745	2016		25.71
Bipolar disorder		[23]	20,352	31,358	2019		23.96
Eating Disorders		[24]	16,992	55,525	2019		25.92

### Instrumental variable selection

We identified statistically significant SNPs from GWAS dataset using a stringent threshold of *p* < 5 × 10^–8^. In cases where an insufficient number of significant SNPs were available, we relaxed the threshold to* p* < 5 × 10^–6^ to ensure a comprehensive inclusion of relevant SNPs [25]. In order to ensure the independence of IVs, we applied a linkage disequilibrium (LD) threshold of *r*^2^ < 0.001 and clump distance > 10,000 kb [26]. Additionally, we employed the F-statistic to evaluate the strength of the IVs and excluded weak IVs to minimize potential bias. IVs with an F-statistic greater than 10 were considered to be strongly associated with the exposure factors [27].

### Statistical analyses

To identify and exclude SNPs associated with potential confounding factors at a genome-wide level, we conducted an investigation using PhenoScanner (www.phenoscanner.medschl.cam.ac.uk) to assess the relationship between these SNPs and the potential risk factors [28]. If any of these SNPs were found to be associated with potential risk factors, they were excluded from further analysis.

The MR-PRESSO test was employed to identify and correct for horizontal pleiotropic outliers, ensuring the robustness of the results [29]. To assess the causal association between female infertility and psychiatric disorders, we utilized three main approaches: the inverse variance weighting (IVW) method, the weighted median approach, and the MR-Egger approach. The IVW method was the primary analytical approach, while the MR-Egger and weighted median methods were used as complementary approaches known for producing more reliable estimates across diverse scenarios, although with reduced efficiency leading to wider confidence intervals. In case of discrepancies among estimates obtained from these approaches, a stricter *p*-value threshold would be applied [29]. Heterogeneity was assessed using Cochran's Q test, and potential horizontal pleiotropy was evaluated through the MR-Egger intercept test and leave-one-out analyses. Furthermore, a funnel plot was employed to assess potential directional pleiotropy.

The analyses were conducted using the TwoSampleMR package (version 0.5.6), MRPRESSO (version 1.0), and SMR (version 1.3.1) in R software (version 4.2.2). The results are presented as odds ratios (ORs) with their corresponding 95% confidence intervals (CIs). Two-sided *p*-values were used, and statistical significance was determined at *p* < 0. 05.

## Results

### Exposure and outcome

To address the potential influence of confounding risk factors on the obtained estimate, we conducted a SNP lookup using Phenoscanner. We considered various factors, including weight, smoking, alcohol, treatment with levothyroxine sodium, self-reported hypothyroidism or myxoedema, thyroid peroxidase antibody positivity, treatment with insulin product, self-reported systemic lupus erythematosis or SLE, age at menarche, and age-started oral contraceptive pill, as potential confounders of female infertility. A total of 26 SNPs associated with these confounding factors were excluded from the analysis. The specific SNPs excluded are as follows: rs10149470, rs1045430, rs1095626, rs11135349, rs113188507, rs12967855, rs1343605, rs1890946, rs200949, rs2509805, rs2568958, rs301799, rs30266, rs3099439, rs34488670, rs3823624, rs56887639, rs5995992, rs61902811, rs6783233, rs7241572, rs7685686, rs111444407, rs174592, rs329319, and rs58990403. Detailed information about these confounders is provided in (Table S1). No potential confounding factors were identified in psychiatric disorders-related SNPs.

A total of 62 SNPs associated with psychiatric disorders (as shown in Table S2) and 85 SNPs associated with female infertility (as shown in Table S3) were extracted as instrumental variables (IVs). All IVs had *F*-statistics exceeding the standard threshold of 10, indicating robust instruments [24]. It is important to note that there was no overlap between the variables of interest, namely psychiatric disorders and female infertility.

### The causal effect of psychiatric disorders on female infertility

**Fig. 2:**
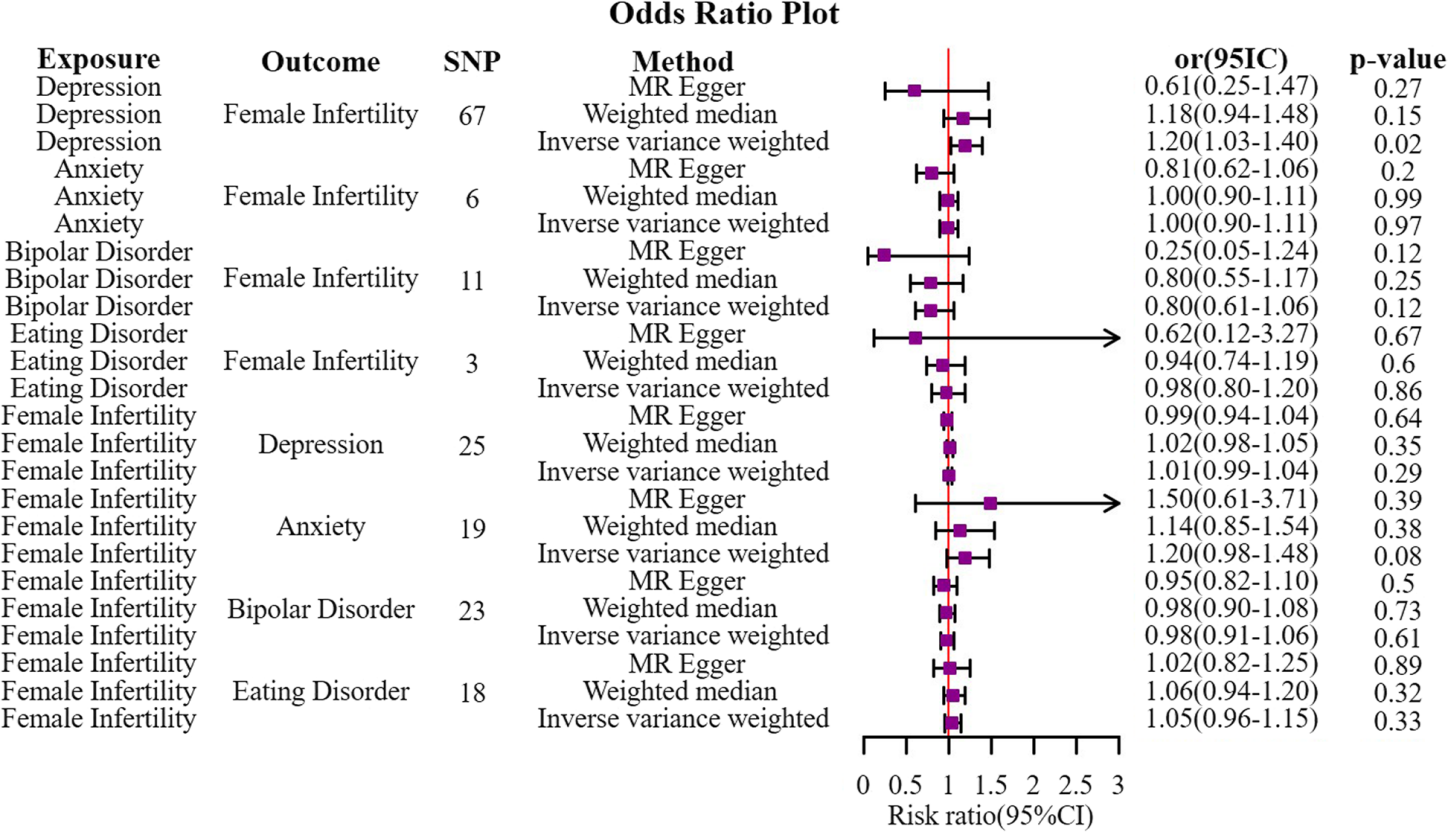
Forest plot of MR analysis between exposure and outcome

Figure 2 presents the primary results of MR estimates for psychiatric disorders. The Cochran's Q test and MR-PRESSO test revealed heterogeneity among the five SNPs associated with anxiety, while other IVs for psychiatric disorders did not exhibit heterogeneity (refer to Tables S4 and S5).

The IVW approach was utilized as the primary analysis method. The results indicated that depression may have a contributing role in female infertility based on the IVW approach (OR = 1.23, 95% CI = 1.00‐1.51, *p* = 0.04). The weighted median approach yielded similar risk estimates (OR = 1.37, 95% CI = 1.05–1.79, *p* = 0.02). On the other hand, weak evidence suggested that bipolar disorder does not increase the risk of female infertility according to the IVW approach (OR = 0.74, 95% CI = 0.56‐0.99, *p* = 0.049). Furthermore, the results of the MR-Egger intercept test (refer to Table S6) indicated the absence of directional pleiotropy for depression (egger-intercept = 0.025, se = 0.015, *p* = 0.11) and bipolar disorder (egger-intercept = 0.047, se = 0.033, *p* = 0.18). Additionally, no single SNP strongly violated the overall effect of depression and bipolar disorder on female infertility in the leave-one-out sensitivity analysis (refer to Figures S1 and S3).

Moreover, we found no causal association between anxiety (OR = 1.00, 95% CI = 0.88‐1.13, *p* = 0.96) and eating disorder (OR = 0.98, 95% CI = 0.80‐1.20, *p* = 0.86) with female infertility according to the IVW approach. Although no substantial evidence of a significant intercept (refer to Table S6) was observed for IVs of anxiety (egger-intercept = 0.041, se = 0.029, *p* = 0.26) and eating disorder (egger-intercept = 0.040, se = 0.072, *p* = 0.68), indicating the absence of directional pleiotropy. In leave-one-out sensitivity analyses, the results demonstrated that no single SNPs had a drastic impact on the results after their exclusion in the IVs of eating disorders. However, SNP rs28373923 and rs739315 strongly violated the overall effect of anxiety when eliminated (refer to Figures S2 and S4).

### The causal effect of female infertility on psychiatric disorders

We conducted a MR analysis to estimate the causal effects of female infertility on psychiatric disorders, as illustrated in Fig. 2. The results of the study indicate that there is no causal association between female infertility and depression (OR = 1.01, 95% CI = 0.99–1.04, *p* = 0.29), anxiety (OR = 1.20, 95% CI = 0.98–1.48, *p* = 0.08), bipolar disorder (OR = 0.98, 95% CI = 0.91–1.06, *p* = 0.98), and eating disorder (OR = 1.05, 95% CI = 0.96–1.15, *p* = 0.33) based on the IVW approach.

The Cochran's Q test and MR-PRESSO test revealed no significant heterogeneity in the effects among the SNPs associated with female infertility (*p* > 0.05, refer to Tables S4 and S5). Additionally, the MR-Egger intercept estimator indicated the absence of horizontal pleiotropy among the IVs (refer to Table S6). In the leave-one-out sensitivity analyses, no single SNP strongly violated the overall effect of female infertility on psychiatric disorders (refer to Figures S5-6).

## Discussion

We conducted a MR analysis to investigate the causal relationship between female infertility and psychiatric disorders. Our analysis yielded important findings regarding the impact of specific psychiatric disorders on the risk of female infertility. Specifically, we found evidence supporting the adverse effect of depression on the risk of infertility in women. On the other hand, our analysis indicated that bipolar disorder does not increase the risk of infertility in women. However, our analysis did not reveal any evidence supporting a causal relationship between anxiety disorders and eating disorders on the risk of female infertility.

Previous observational studies have suggested a potential association between female infertility and psychiatric disorders [6–9]. and our MR study provides support for some of these findings. Specifically, depression, a complex condition affecting both physical and mental well-being, has been found to be prevalent among infertile women. Prior research has indicated that depression may act as a risk factor for female infertility through various mechanisms, including the suppression of the HPOA, oocyte damage, and apoptosis of ovarian granulosa cells. Studies have highlighted the involvement of the hypothalamic–pituitary–adrenal (HPA) axis in depression, demonstrating abnormal activation of the HPA axis and subsequent release of corticotropin-releasing hormone (CRH). Elevated levels of CRH have been found to inhibit the HPOA by suppressing hypothalamic gonadotropin-releasing hormone (GnRH) release and directly affecting the ovary [30, 31]. Animal studies have shown that CRH can inhibit hypothalamic luteinizing hormone (LH) and stimulate follicle-stimulating hormone (FSH) release in female rats [30]. Additionally, CRH has been found to inhibit estrogen (E_2_) production in rat and human granulosa cells [31]. Moreover, CRH induces increased glucocorticoid secretion from the adrenal cortex, and glucocorticoids have been shown to have detrimental effects on oocytes [32]. Experimental studies in female mice have demonstrated that exogenous glucocorticoid injections impair oocyte developmental potential and trigger apoptosis of ovarian granulosa cells and oocytes [33].

Bipolar disorder, another physical disorder, has been associated with lower fertility rates and a higher incidence of infertility in women according to previous research [14, 34]. Women with bipolar disorder often experience menstrual cycle disturbances [35], suggesting an imbalance in steroidal gonadotropic hormones, including reduced levels of estrogen and progesterone and increased levels of testosterone [36, 37]. However, our MR study findings indicate that bipolar disorder is not a risk factor for female infertility. This contradictory result may be attributed to confounding factors present in previous clinical studies and the potential influence of psychiatric medications, such as valproate, on the risk of polycystic ovary syndrome or the abnormal regulation of female steroid hormones due to lithium treatment [38, 39]. Further research is warranted to validate the relationship between bipolar disorder and female infertility.

Our study possesses several notable strengths. Firstly, we employed the MR design, which helps minimize the risk of reverse causation and confounding biases inherent in observational studies. Secondly, we focused on cohorts comprising individuals of European descent to mitigate potential confounding related to population stratification. Lastly, the lower likelihood of exposure to psychiatric disorders among our study subjects enhances the internal validity of our findings.

However, several limitations of our study should be acknowledged. Firstly, the generalizability of our findings to other populations is uncertain since all GWAS data used in our analysis are derived from European populations. Future investigations should include more diverse samples to assess the transferability of phenotypic and genetic associations across ancestry groups. Secondly, to increase the number of SNPs for assessing the causal effects of psychiatric disorders, we utilized a relatively lenient *p*-value threshold of 5 × 10^–6^, potentially incorporating SNPs with weaker associations and smaller effect sizes. Future studies should consider employing stricter *p*-value thresholds to enhance the reliability and reproducibility of findings. Lastly, although our findings suggest a potential association between depression and female infertility, the lack of relevant data prevented us from conducting a subtype analysis of depression.

To address these limitations, future research endeavors should encompass larger and more diverse datasets, incorporate multiple GWAS databases, and employ stricter *p*-value thresholds to ensure robust and generalizable findings.

## Conclusion

The results of our investigation provided limited evidence to support the hypothesis that depression may increase the susceptibility to female infertility. Additionally, our findings indicated that bipolar disorder is not associated with an increased risk of female infertility. These findings imply that interventions targeting emotional well-being could potentially have a positive impact on the prevention and treatment of female infertility. The disparity between our findings and traditional observational studies may be attributed to inherent limitations, including the possibility of reverse causation or residual confounding. Notably, our study offers a novel perspective by employing MR analysis, which effectively addresses these limitations and yields compelling evidence supporting a potential causal relationship between depression and female infertility.

### Supplementary Information

**Additional file 1: Table S1.** Psychiatric disorders-related IVs trait as potential confounders of female infertility in PhenoScanner V2. **Table S2.** Psychiatric disorders associated with female infertility. **Table S3.** Female infertility associated with psychiatric disorders. **Table S4.** The Cochran's Q test for exposure on outcome. **Table S5.** The MR-PRESSO test for exposure on outcome. **Table S6.** The MR-Egger intercept test for exposure on outcome.

**Additional file 2: Figure S1.** MR analysis for depression on female infertility. (a) Scatter plots from depression on female infertility (b) Funnel plot from depression on female infertility (c) Forest plot from depression on female infertility (d) Leave-one-out plot from depression on female infertility. **Figure S2. **MR analysis for anxiety on female infertility (a) Scatter plots from anxiety on female infertility (b) Funnel plot from anxiety on female infertility; (c) Forest plot from anxiety on female infertility (d) Leave-one-out plot from anxiety on female infertility. **Figure S3.** MR analysis for bipolar disorder on female infertility (a) Scatter plots from bipolar disorder on female infertility; (b) Funnel plot from bipolar disorder on female infertility (c) Forest plot from bipolar disorder on female infertility (d) Leave-one-out plot from bipolar disorder on female infertility. **Figure S4.** MR analysis for eating disorders on female infertility (a) Scatter plots from eating disorders on female infertility; (b) Funnel plot from eating disorders on female infertility. (c) Forest plot from eating disorders on female infertility (d) Leave-one-out plot from eating disorders on female infertility. **Figure S5.** MR analysis for female infertility on depression (a) Scatter plots from female infertility on depression; (b) Funnel plot from female infertility on depression (c) Forest plot from female infertility on depression (d) Leave-one-out plot from female infertility on depression. **Figure S6.** MR analysis for female infertility on anxiety (a) Scatter plots from female infertility on anxiety (b) Funnel plot from female infertility on anxiety; (c) Forest plot from female infertility on anxiety (d) Leave-one-out plot from female infertility on anxiety. **Figure S7.** MR analysis for female infertility on bipolar disorder (a) Scatter plots from female infertility on bipolar disorder; (b) Funnel plot from female infertility on bipolar disorder; (c) Forest plot from female infertility on bipolar disorder;(d) Leave-one-out plot from female infertility on bipolar disorder. **Figure S8.** MR analysis for female infertility on eating disorders. (a) Scatter plots from female infertility on eating disorders; (b) Funnel plot from female infertility on eating disorders; (c) Forest plot from female infertility on eating disorders (d) Leave-one-out plot from female infertility on eating disorders.

## Data Availability

The datasets analyzed during the current study are available in the FinnGen GWAS Summary Statistics R8 release (https://r8.finngen.fi/) and the Psychiatric Genomics Consortium (https://pgc.unc.edu/about-us/.
