# Supplementary material for: The causal relationship of female infertility and psychiatric disorders in the European population: a bidirectional two-sample Mendelian randomization study
Source: BMC Womens Health. 2024 Jan 19;24:54. doi: 10.1186/s12905-024-02888-5 (PMC10797979; doi:10.1186/s12905-024-02888-5)
Supplement: Supplementary file 1 — Additional file 1: Table S1. Psychiatric disorders-related IVs trait as potential confounders of female infertility in PhenoScanner V2. Table S2. Psychiatric disorders associated with female infertility. Table S3. Female infertility associated with psychiatric disorders. Table S4. The Cochran's Q test for exposure on outcome. Table S5. The MR-PRESSO test for exposure on outcome. Table S6. The MR-Egger intercept test for exposure on outcome. [file 12905_2024_2888_MOESM1_ESM.docx]

**Table S1. psychiatric disorders-related IVs trait as potential confounders of female infertility in PhenoScanner V2**

| exposure | SNP | effect allele | other allele | trait | beta | SE | p-value | sample size |
| --- | --- | --- | --- | --- | --- | --- | --- | --- |
| Depression | rs34488670 | C | T | Current tobacco smoking | 0.008902 | 0.001622 | 4.079E-08 | 337030 |
| Depression | rs34488670 | C | T | Past tobacco smoking | -0.02106 | 0.003891 | 6.217E-08 | 310749 |
| Depression | rs56887639 | A | G | Past tobacco smoking | 0.01656 | 0.003597 | 0.000004166 | 310749 |
| Depression | rs5995992 | C | T | Current tobacco smoking | -0.006872 | 0.001462 | 0.000002609 | 337030 |
| Depression | rs12967855 | A | G | Current tobacco smoking | 0.006941 | 0.001411 | 8.743E-07 | 337030 |
| Depression | rs12967855 | A | G | Pack years adult smoking as proportion of life span exposed to smoking | 0.02143 | 0.004672 | 0.000004513 | 101726 |
| Depression | rs1890946 | C | T | Nicotine dependence smoking cigarettes per day | NA | NA | 0.0000014 | 31266 |
| Depression | rs200949 | A | G | Why stopped smoking: health precaution | 0.01528 | 0.00342 | 0.000007953 | 82082 |
| Depression | rs2568958 | A | G | Body mass index adjusted for smoking in females | 0.0223 | 0.0046 | 0.000001126 | 114994 |
| Depression | rs2568958 | A | G | Body mass index adjusted for smoking | 0.0281 | 0.0037 | 1.38E-14 | 186113 |
| Depression | rs2568958 | A | G | Body mass index adjusted for smoking | 0.0263 | 0.0034 | 1.297E-14 | 216490 |
| Depression | rs3823624 | C | T | Weight | -0.01508 | 0.002773 | 5.357E-08 | 336227 |
| Depression | rs113188507 | A | G | Weight | 0.01155 | 0.002322 | 6.474E-07 | 336227 |
| Depression | rs200949 | A | G | Weight | -0.01568 | 0.003022 | 2.105E-07 | 336227 |
| Depression | rs2568958 | A | G | Overweight | 0.062 | 0.0092 | 1.1E-11 | 158401 |
| Depression | rs2568958 | A | G | Weight in females | 0.032 | 0.0063 | 0.000000463 | 67572 |
| Depression | rs2568958 | A | G | Weight | 0.03438 | 0.004557 | 4.522E-14 | 125882 |
| Depression | rs2568958 | A | G | Weight | NA | NA | 0.000000021 | 34416 |
| Depression | rs2568958 | A | G | Weight | 3.37 | 0.6005 | 0.00000002 | - |
| Depression | rs2568958 | A | G | Weight | 0.01878 | 0.002163 | 3.841E-18 | 336227 |
| Depression | rs2568958 | A | G | Body weight | NA | NA | 0.00000002 | - |
| Depression | rs3099439 | T | C | Alcohol intake frequency | 0.02025 | 0.003586 | 1.631E-08 | 336965 |
| Depression | rs3823624 | C | T | Alcohol intake frequency | -0.02178 | 0.004642 | 0.00000271 | 336965 |
| Depression | rs56887639 | A | G | Alcohol intake frequency | 0.02105 | 0.004036 | 1.835E-07 | 336965 |
| Depression | rs61902811 | A | G | Alcohol intake frequency | 0.01851 | 0.003681 | 4.947E-07 | 336965 |
| Depression | rs6783233 | C | T | Alcohol intake frequency | -0.01821 | 0.003944 | 0.000003877 | 336965 |
| Depression | rs7685686 | A | G | Alcohol intake frequency | 0.02012 | 0.0036 | 2.288E-08 | 336965 |
| Depression | rs12967855 | A | G | Alcohol usually taken with meals | -0.007759 | 0.001658 | 0.000002856 | 172454 |
| Depression | rs2509805 | C | T | Alcohol intake frequency | -0.01807 | 0.003814 | 0.000002167 | 336965 |
| Depression | rs2509805 | C | T | Alcohol usually taken with meals | 0.007601 | 0.001666 | 0.000005037 | 172454 |
| Depression | rs2568958 | A | G | Alcohol intake versus 10 years previously | 0.00942 | 0.001881 | 5.471E-07 | 313248 |
| Depression | rs200949 | A | G | Treatment with levothyroxine sodium | -0.00522 | 0.0006805 | 1.707E-14 | 337159 |
| Depression | rs200949 | A | G | Self-reported hypothyroidism or myxoedema | -0.005155 | 0.0007384 | 2.949E-12 | 337159 |
| Depression | rs301799 | C | T | Thyroid peroxidase antibody positivity | 0.1044 | 0.02234 | 0.000003 | - |
| Depression | rs200949 | A | G | Treatment with insulin product | -0.002571 | 0.0003424 | 6.009E-14 | 337159 |
| Depression | rs200949 | A | G | Self-reported systemic lupus erythematosis or sle | -0.0005908 | 0.0001142 | 0.000000232 | 337159 |
| Depression | rs6783233 | C | T | Age at menarche | 0.01785 | 0.002748 | 8.385E-11 | 176008 |
| Depression | rs6783233 | C | T | Age at menarche | 0.04 | 0.005603 | 9.4E-13 | 182416 |
| Depression | rs2568958 | A | G | Age at menarche | -0.01808 | 0.002526 | 8.099E-13 | 176008 |
| Depression | rs2568958 | A | G | Age at menarche | -0.034 | 0.004896 | 3.8E-12 | 182416 |
| Depression | rs301799 | C | T | Age at menarche | -0.023 | 0.00516 | 0.0000083 | 182416 |
| Depression | rs7241572 | A | G | Age-started oral contraceptive pill | -0.0229 | 0.004629 | 7.559E-07 | 144113 |
| Anxiety | rs58990403 | A | G | Weight | 0.01285 | 0.002766 | 0.000003371 | 336227 |
| Bipolar Disorder | rs174592 | G | A | Treatment with levothyroxine sodium | -0.002746 | 0.0004951 | 2.924E-08 | 337159 |
| Bipolar Disorder | rs174592 | G | A | Self-reported hypothyroidism or myxoedema | -0.00272 | 0.0005373 | 4.127E-07 | 337159 |
| Bipolar Disorder | rs329319 | A | G | Age at menarche | 0.0129 | 0.002597 | 6.747E-07 | 176008 |

**Table S2.** **Psychiatric disorders associated with female infertility**

| exposure | outcome | SNP | effect allele | other allele | eaf | beta | se | *p*-value | sample size |
| --- | --- | --- | --- | --- | --- | --- | --- | --- | --- |
| Depression | Female Infertility | rs1002656 | T | C | 0.7033 | -0.0266 | 0.0038 | 2.56E-12 | 807,553 |
| Depression | Female Infertility | rs10061069 | C | G | 0.2212 | -0.0275 | 0.0042 | 5.85E-11 | 807,553 |
| Depression | Female Infertility | rs10149470 | A | G | 0.4869 | -0.0267 | 0.0035 | 2.37E-14 | 807,553 |
| Depression | Female Infertility | rs1021363 | A | G | 0.3547 | 0.0303 | 0.0037 | 2.63E-16 | 807,553 |
| Depression | Female Infertility | rs1045430 | T | G | 0.4792 | -0.0253 | 0.0035 | 4.88E-13 | 807,553 |
| Depression | Female Infertility | rs10774600 | T | C | 0.1656 | -0.0267 | 0.0048 | 2.66E-08 | 807,553 |
| Depression | Female Infertility | rs10789214 | T | C | 0.5661 | 0.0193 | 0.0035 | 3.50E-08 | 807,553 |
| Depression | Female Infertility | rs10817969 | T | G | 0.7173 | 0.0261 | 0.0039 | 2.20E-11 | 807,553 |
| Depression | Female Infertility | rs10913112 | T | C | 0.3767 | -0.0264 | 0.0036 | 2.24E-13 | 807,553 |
| Depression | Female Infertility | rs1095626 | T | C | 0.5799 | -0.0264 | 0.0035 | 4.60E-14 | 807,553 |
| Depression | Female Infertility | rs11135349 | A | C | 0.4713 | -0.0295 | 0.0035 | 3.50E-17 | 807,553 |
| Depression | Female Infertility | rs113188507 | A | G | 0.2838 | 0.0221 | 0.0039 | 1.46E-08 | 807,553 |
| Depression | Female Infertility | rs1152578 | T | C | 0.4357 | -0.0218 | 0.0035 | 4.71E-10 | 807,553 |
| Depression | Female Infertility | rs11579246 | A | G | 0.9067 | 0.0381 | 0.0061 | 4.21E-10 | 807,553 |
| Depression | Female Infertility | rs1226412 | T | C | 0.7917 | 0.0256 | 0.0043 | 2.62E-09 | 807,553 |
| Depression | Female Infertility | rs12967143 | C | G | 0.6984 | -0.0312 | 0.0038 | 2.20E-16 | 807,553 |
| Depression | Female Infertility | rs12967855 | A | G | 0.3295 | 0.0265 | 0.0037 | 7.94E-13 | 807,553 |
| Depression | Female Infertility | rs1343605 | A | C | 0.384 | 0.0313 | 0.0036 | 3.49E-18 | 807,553 |
| Depression | Female Infertility | rs1409379 | T | C | 0.7641 | 0.0249 | 0.0041 | 1.25E-09 | 807,553 |
| Depression | Female Infertility | rs141954845 | A | G | 0.388 | 0.0229 | 0.0037 | 6.05E-10 | 807,553 |
| Depression | Female Infertility | rs143186028 | T | G | 0.1778 | 0.0277 | 0.0046 | 1.73E-09 | 807,553 |
| Depression | Female Infertility | rs1448938 | A | G | 0.4171 | 0.0214 | 0.0035 | 9.70E-10 | 807,553 |
| Depression | Female Infertility | rs1466887 | T | C | 0.5511 | -0.0199 | 0.0036 | 3.24E-08 | 807,553 |
| Depression | Female Infertility | rs1568452 | T | C | 0.3851 | 0.0248 | 0.0036 | 5.62E-12 | 807,553 |
| Depression | Female Infertility | rs17641524 | T | C | 0.2091 | -0.032 | 0.0043 | 9.93E-14 | 807,553 |
| Depression | Female Infertility | rs1890946 | T | C | 0.4671 | -0.0235 | 0.0035 | 1.89E-11 | 807,553 |
| Depression | Female Infertility | rs1982277 | T | C | 0.7594 | 0.0279 | 0.0041 | 1.01E-11 | 807,553 |
| Depression | Female Infertility | rs198457 | T | C | 0.1925 | -0.0292 | 0.0046 | 2.18E-10 | 807,553 |
| Depression | Female Infertility | rs200949 | A | G | 0.8744 | 0.048 | 0.0053 | 1.35E-19 | 807,553 |
| Depression | Female Infertility | rs2043539 | A | G | 0.4177 | 0.0273 | 0.0035 | 6.19E-15 | 807,553 |
| Depression | Female Infertility | rs2509805 | T | C | 0.3209 | 0.022 | 0.0038 | 7.06E-09 | 807,553 |
| Depression | Female Infertility | rs2568958 | A | G | 0.6156 | 0.0373 | 0.0036 | 3.73E-25 | 807,553 |
| Depression | Female Infertility | rs301799 | T | C | 0.5694 | -0.025 | 0.0035 | 9.14E-13 | 807,553 |
| Depression | Female Infertility | rs30266 | A | G | 0.3296 | 0.0308 | 0.0037 | 8.48E-17 | 807,553 |
| Depression | Female Infertility | rs3099439 | T | C | 0.5288 | -0.0276 | 0.0035 | 3.13E-15 | 807,553 |
| Depression | Female Infertility | rs3213572 | A | G | 0.4745 | 0.0217 | 0.0035 | 5.65E-10 | 807,553 |
| Depression | Female Infertility | rs33431 | T | C | 0.6144 | 0.0198 | 0.0036 | 3.80E-08 | 807,553 |
| Depression | Female Infertility | rs34488670 | T | C | 0.7887 | -0.0252 | 0.0043 | 4.62E-09 | 807,553 |
| Depression | Female Infertility | rs34937911 | T | C | 0.8838 | 0.0304 | 0.0055 | 3.25E-08 | 807,553 |
| Depression | Female Infertility | rs3793577 | A | G | 0.4665 | -0.0229 | 0.0035 | 6.04E-11 | 807,553 |
| Depression | Female Infertility | rs3823624 | T | C | 0.8067 | 0.0272 | 0.0045 | 1.50E-09 | 807,553 |
| Depression | Female Infertility | rs4346585 | T | C | 0.696 | -0.0236 | 0.0038 | 5.28E-10 | 807,553 |
| Depression | Female Infertility | rs45510091 | A | G | 0.9472 | 0.0448 | 0.008 | 2.14E-08 | 807,553 |
| Depression | Female Infertility | rs4772087 | T | C | 0.3732 | 0.0227 | 0.0036 | 2.87E-10 | 807,553 |
| Depression | Female Infertility | rs56314503 | T | G | 0.7487 | -0.0254 | 0.004 | 2.15E-10 | 807,553 |
| Depression | Female Infertility | rs56887639 | A | G | 0.7264 | -0.0278 | 0.0039 | 1.02E-12 | 807,553 |
| Depression | Female Infertility | rs57344483 | A | G | 0.9259 | -0.038 | 0.0068 | 2.29E-08 | 807,553 |
| Depression | Female Infertility | rs58104186 | A | G | 0.4689 | 0.0237 | 0.0035 | 1.28E-11 | 807,553 |
| Depression | Female Infertility | rs5995992 | T | C | 0.7155 | -0.0266 | 0.0039 | 9.07E-12 | 807,553 |
| Depression | Female Infertility | rs60157091 | T | C | 0.515 | 0.02 | 0.0035 | 1.10E-08 | 807,553 |
| Depression | Female Infertility | rs61902811 | A | G | 0.3682 | -0.0257 | 0.0036 | 9.41E-13 | 807,553 |
| Depression | Female Infertility | rs61990288 | A | G | 0.5083 | -0.026 | 0.0035 | 1.10E-13 | 807,553 |
| Depression | Female Infertility | rs6783233 | T | C | 0.2833 | 0.0218 | 0.0039 | 2.27E-08 | 807,553 |
| Depression | Female Infertility | rs7030813 | T | C | 0.3736 | 0.0253 | 0.0036 | 2.10E-12 | 807,553 |
| Depression | Female Infertility | rs7200826 | T | C | 0.2551 | 0.028 | 0.004 | 2.56E-12 | 807,553 |
| Depression | Female Infertility | rs7241572 | A | G | 0.201 | 0.028 | 0.0044 | 1.97E-10 | 807,553 |
| Depression | Female Infertility | rs725616 | T | C | 0.3644 | 0.0204 | 0.0036 | 1.46E-08 | 807,553 |
| Depression | Female Infertility | rs75581564 | A | G | 0.1165 | 0.0301 | 0.0054 | 2.49E-08 | 807,553 |
| Depression | Female Infertility | rs7585722 | T | C | 0.8458 | -0.0269 | 0.0048 | 2.09E-08 | 807,553 |
| Depression | Female Infertility | rs7659414 | A | C | 0.5782 | -0.0201 | 0.0035 | 9.31E-09 | 807,553 |
| Depression | Female Infertility | rs7685686 | A | G | 0.5753 | 0.0202 | 0.0036 | 2.01E-08 | 807,553 |
| Depression | Female Infertility | rs78337797 | T | G | 0.8781 | 0.0306 | 0.0055 | 2.64E-08 | 807,553 |
| Depression | Female Infertility | rs7837935 | T | G | 0.1522 | -0.0292 | 0.0049 | 2.54E-09 | 807,553 |
| Depression | Female Infertility | rs7932640 | T | C | 0.4417 | 0.0281 | 0.0035 | 9.86E-16 | 807,553 |
| Depression | Female Infertility | rs8037355 | T | C | 0.5556 | -0.0233 | 0.0035 | 2.79E-11 | 807,553 |
| Depression | Female Infertility | rs9592461 | A | G | 0.4874 | 0.0216 | 0.0035 | 6.77E-10 | 807,553 |
| Depression | Female Infertility | rs997934 | T | C | 0.3795 | 0.0198 | 0.0036 | 3.8E-08 | 807,553 |
| Anxiety | Female Infertility | rs1709393 | T | C | 0.5793 | -0.1509 | 0.0267 | 1.59E-08 | 21,761 |
| Anxiety | Female Infertility | rs2146346 | A | G | 0.5869 | 0.1441 | 0.0298 | 1.33E-06 | 21,761 |
| Anxiety | Female Infertility | rs2753188 | A | G | 0.7268 | 0.1603 | 0.0332 | 1.38E-06 | 21,761 |
| Anxiety | Female Infertility | rs28373923 | A | G | 0.0675 | 0.4193 | 0.0915 | 4.59E-06 | 21,761 |
| Anxiety | Female Infertility | rs58990403 | A | G | 0.792 | -0.1851 | 0.0391 | 2.2E-06 | 21,761 |
| Anxiety | Female Infertility | rs739315 | A | G | 0.5688 | -0.1537 | 0.0329 | 2.99E-06 | 21,761 |
| Bipolar Disorder | Female Infertility | rs10744560 | T | C | 0.352 | 0.036134 | 0.014 | 0.009852 | 51,710 |
| Bipolar Disorder | Female Infertility | rs111444407 | T | C | 0.165 | 0.050639 | 0.0184 | 0.005921 | 51,710 |
| Bipolar Disorder | Female Infertility | rs11724116 | T | C | 0.148 | -0.04521 | 0.0188 | 0.016187 | 51,710 |
| Bipolar Disorder | Female Infertility | rs13231398 | C | G | 0.101 | -0.05242 | 0.0219 | 0.016685 | 51,710 |
| Bipolar Disorder | Female Infertility | rs138321 | A | G | 0.522 | 0.03444 | 0.0135 | 0.010738 | 51,710 |
| Bipolar Disorder | Female Infertility | rs174592 | A | G | 0.618 | -0.03361 | 0.0141 | 0.017126 | 51,710 |
| Bipolar Disorder | Female Infertility | rs329319 | A | G | 0.444 | 0.034223 | 0.0139 | 0.013812 | 51,710 |
| Bipolar Disorder | Female Infertility | rs55648125 | A | G | 0.885 | -0.05085 | 0.0215 | 0.018015 | 51,710 |
| Bipolar Disorder | Female Infertility | rs73496688 | A | T | 0.157 | 0.047209 | 0.019 | 0.012967 | 51,710 |
| Bipolar Disorder | Female Infertility | rs884301 | T | C | 0.392 | 0.034873 | 0.0138 | 0.011503 | 51,710 |
| Bipolar Disorder | Female Infertility | rs9834970 | T | C | 0.483 | -0.04387 | 0.0134 | 0.001062 | 51,710 |
| Eating Disorders | Female Infertility | rs11165643 | T | C | NA | 0.074699 | 0.0135 | 3.14E-08 | 72,517 |
| Eating Disorders | Female Infertility | rs2287348 | T | C | NA | -0.10441 | 0.0179 | 5.45E-09 | 72,517 |
| Eating Disorders | Female Infertility | rs9874207 | C | T | NA | -0.0813 | 0.0145 | 2.06E-08 | 72,517 |

**Table S3. Female infertility associated with psychiatric disorders**

| exposure | outcome | SNP | effect allele | other allele | eaf | beta | se | *p*-value | sample size |
| --- | --- | --- | --- | --- | --- | --- | --- | --- | --- |
| Female Infertility | Depression | rs10458884 | C | T | 0.537029 | 0.065361 | 0.014092 | 3.52E-06 | 807,553 |
| Female Infertility | Depression | rs10503673 | G | A | 0.804836 | 0.088425 | 0.018174 | 1.14E-06 | 807,553 |
| Female Infertility | Depression | rs10512623 | T | C | 0.20241 | 0.080698 | 0.017291 | 3.06E-06 | 807,553 |
| Female Infertility | Depression | rs10954374 | A | G | 0.91728 | -0.11352 | 0.024832 | 4.84E-06 | 807,553 |
| Female Infertility | Depression | rs11056435 | A | G | 0.197031 | 0.092216 | 0.017396 | 1.15E-07 | 807,553 |
| Female Infertility | Depression | rs11073495 | A | G | 0.775132 | 0.083213 | 0.017146 | 1.21E-06 | 807,553 |
| Female Infertility | Depression | rs112691364 | A | G | 0.029486 | -0.23111 | 0.044153 | 1.66E-07 | 807,553 |
| Female Infertility | Depression | rs114903342 | A | G | 0.003775 | 0.479847 | 0.104398 | 4.3E-06 | 807,553 |
| Female Infertility | Depression | rs115338272 | C | T | 0.039841 | 0.159983 | 0.034637 | 3.86E-06 | 807,553 |
| Female Infertility | Depression | rs12806135 | G | A | 0.061973 | -0.14442 | 0.030178 | 1.7E-06 | 807,553 |
| Female Infertility | Depression | rs13028479 | T | G | 0.594588 | 0.067009 | 0.014407 | 3.3E-06 | 807,553 |
| Female Infertility | Depression | rs1424035 | A | C | 0.005481 | 0.457065 | 0.085265 | 8.3E-08 | 807,553 |
| Female Infertility | Depression | rs190946682 | A | G | 0.062927 | 0.198704 | 0.027811 | 9.02E-13 | 807,553 |
| Female Infertility | Depression | rs236332 | C | T | 0.246088 | 0.078294 | 0.016139 | 1.23E-06 | 807,553 |
| Female Infertility | Depression | rs2779747 | T | G | 0.577866 | -0.06692 | 0.014177 | 2.35E-06 | 807,553 |
| Female Infertility | Depression | rs313980 | A | C | 0.80806 | -0.0812 | 0.017652 | 4.22E-06 | 807,553 |
| Female Infertility | Depression | rs55938609 | C | G | 0.164166 | 0.094599 | 0.018644 | 3.9E-07 | 807,553 |
| Female Infertility | Depression | rs584336 | A | G | 0.299812 | 0.074614 | 0.015235 | 9.7E-07 | 807,553 |
| Female Infertility | Depression | rs58924196 | G | C | 0.066249 | 0.134099 | 0.027461 | 1.04E-06 | 807,553 |
| Female Infertility | Depression | rs6025 | T | C | 0.020732 | -0.24048 | 0.052434 | 4.51E-06 | 807,553 |
| Female Infertility | Depression | rs62010525 | G | C | 0.269497 | 0.072023 | 0.01572 | 4.61E-06 | 807,553 |
| Female Infertility | Depression | rs6835215 | G | A | 0.008409 | 0.363577 | 0.072572 | 5.45E-07 | 807,553 |
| Female Infertility | Depression | rs73527055 | C | T | 0.443192 | -0.0683 | 0.014213 | 1.55E-06 | 807,553 |
| Female Infertility | Depression | rs77893563 | A | G | 0.023406 | -0.22743 | 0.049285 | 3.94E-06 | 807,553 |
| Female Infertility | Depression | rs78721914 | G | A | 0.058209 | 0.134988 | 0.028992 | 3.22E-06 | 807,553 |
| Female Infertility | Anxiety | rs10458884 | C | T | 0.537029 | 0.065361 | 0.014092 | 3.52E-06 | 21,761 |
| Female Infertility | Anxiety | rs10503673 | G | A | 0.804836 | 0.088425 | 0.018174 | 1.14E-06 | 21,761 |
| Female Infertility | Anxiety | rs10512623 | T | C | 0.20241 | 0.080698 | 0.017291 | 3.06E-06 | 21,761 |
| Female Infertility | Anxiety | rs10954374 | A | G | 0.91728 | -0.11352 | 0.024832 | 4.84E-06 | 21,761 |
| Female Infertility | Anxiety | rs11056435 | A | G | 0.197031 | 0.092216 | 0.017396 | 1.15E-07 | 21,761 |
| Female Infertility | Anxiety | rs11073495 | A | G | 0.775132 | 0.083213 | 0.017146 | 1.21E-06 | 21,761 |
| Female Infertility | Anxiety | rs115338272 | C | T | 0.039841 | 0.159983 | 0.034637 | 3.86E-06 | 21,761 |
| Female Infertility | Anxiety | rs12806135 | G | A | 0.061973 | -0.14442 | 0.030178 | 1.7E-06 | 21,761 |
| Female Infertility | Anxiety | rs13028479 | T | G | 0.594588 | 0.067009 | 0.014407 | 3.3E-06 | 21,761 |
| Female Infertility | Anxiety | rs236332 | C | T | 0.246088 | 0.078294 | 0.016139 | 1.23E-06 | 21,761 |
| Female Infertility | Anxiety | rs2779747 | T | G | 0.577866 | -0.06692 | 0.014177 | 2.35E-06 | 21,761 |
| Female Infertility | Anxiety | rs313980 | A | C | 0.80806 | -0.0812 | 0.017652 | 4.22E-06 | 21,761 |
| Female Infertility | Anxiety | rs479329 | C | T | 0.540994 | -0.06603 | 0.014204 | 3.35E-06 | 21,761 |
| Female Infertility | Anxiety | rs55938609 | C | G | 0.164166 | 0.094599 | 0.018644 | 3.9E-07 | 21,761 |
| Female Infertility | Anxiety | rs584336 | A | G | 0.299812 | 0.074614 | 0.015235 | 9.7E-07 | 21,761 |
| Female Infertility | Anxiety | rs58924196 | G | C | 0.066249 | 0.134099 | 0.027461 | 1.04E-06 | 21,761 |
| Female Infertility | Anxiety | rs62010525 | G | C | 0.269497 | 0.072023 | 0.01572 | 4.61E-06 | 21,761 |
| Female Infertility | Anxiety | rs73527055 | C | T | 0.443192 | -0.0683 | 0.014213 | 1.55E-06 | 21,761 |
| Female Infertility | Anxiety | rs78721914 | G | A | 0.058209 | 0.134988 | 0.028992 | 3.22E-06 | 21,761 |
| Female Infertility | Bipolar Disorder | rs10458884 | C | T | 0.537029 | 0.065361 | 0.014092 | 3.52E-06 | 51,710 |
| Female Infertility | Bipolar Disorder | rs10503673 | G | A | 0.804836 | 0.088425 | 0.018174 | 1.14E-06 | 51,710 |
| Female Infertility | Bipolar Disorder | rs10512623 | T | C | 0.20241 | 0.080698 | 0.017291 | 3.06E-06 | 51,710 |
| Female Infertility | Bipolar Disorder | rs10954374 | A | G | 0.91728 | -0.11352 | 0.024832 | 4.84E-06 | 51,710 |
| Female Infertility | Bipolar Disorder | rs11056435 | A | G | 0.197031 | 0.092216 | 0.017396 | 1.15E-07 | 51,710 |
| Female Infertility | Bipolar Disorder | rs11073495 | A | G | 0.775132 | 0.083213 | 0.017146 | 1.21E-06 | 51,710 |
| Female Infertility | Bipolar Disorder | rs112691364 | A | G | 0.029486 | -0.23111 | 0.044153 | 1.66E-07 | 51,710 |
| Female Infertility | Bipolar Disorder | rs114903342 | A | G | 0.003775 | 0.479847 | 0.104398 | 4.3E-06 | 51,710 |
| Female Infertility | Bipolar Disorder | rs115338272 | C | T | 0.039841 | 0.159983 | 0.034637 | 3.86E-06 | 51,710 |
| Female Infertility | Bipolar Disorder | rs12806135 | G | A | 0.061973 | -0.14442 | 0.030178 | 1.7E-06 | 51,710 |
| Female Infertility | Bipolar Disorder | rs13028479 | T | G | 0.594588 | 0.067009 | 0.014407 | 3.3E-06 | 51,710 |
| Female Infertility | Bipolar Disorder | rs1424035 | A | C | 0.005481 | 0.457065 | 0.085265 | 8.3E-08 | 51,710 |
| Female Infertility | Bipolar Disorder | rs189158115 | G | A | 0.022057 | -0.26969 | 0.052052 | 2.21E-07 | 51,710 |
| Female Infertility | Bipolar Disorder | rs236332 | C | T | 0.246088 | 0.078294 | 0.016139 | 1.23E-06 | 51,710 |
| Female Infertility | Bipolar Disorder | rs2779747 | T | G | 0.577866 | -0.06692 | 0.014177 | 2.35E-06 | 51,710 |
| Female Infertility | Bipolar Disorder | rs313980 | A | C | 0.80806 | -0.0812 | 0.017652 | 4.22E-06 | 51,710 |
| Female Infertility | Bipolar Disorder | rs479329 | C | T | 0.540994 | -0.06603 | 0.014204 | 3.35E-06 | 51,710 |
| Female Infertility | Bipolar Disorder | rs584336 | A | G | 0.299812 | 0.074614 | 0.015235 | 9.7E-07 | 51,710 |
| Female Infertility | Bipolar Disorder | rs6025 | T | C | 0.020732 | -0.24048 | 0.052434 | 4.51E-06 | 51,710 |
| Female Infertility | Bipolar Disorder | rs6835215 | G | A | 0.008409 | 0.363577 | 0.072572 | 5.45E-07 | 51,710 |
| Female Infertility | Bipolar Disorder | rs73527055 | C | T | 0.443192 | -0.0683 | 0.014213 | 1.55E-06 | 51,710 |
| Female Infertility | Bipolar Disorder | rs77893563 | A | G | 0.023406 | -0.22743 | 0.049285 | 3.94E-06 | 51,710 |
| Female Infertility | Bipolar Disorder | rs78721914 | G | A | 0.058209 | 0.134988 | 0.028992 | 3.22E-06 | 51,710 |
| Female Infertility | Eating Disorders | rs10458884 | C | T | 0.537029 | 0.065361 | 0.014092 | 3.52E-06 | 72,517 |
| Female Infertility | Eating Disorders | rs10503673 | G | A | 0.804836 | 0.088425 | 0.018174 | 1.14E-06 | 72,517 |
| Female Infertility | Eating Disorders | rs10512623 | T | C | 0.20241 | 0.080698 | 0.017291 | 3.06E-06 | 72,517 |
| Female Infertility | Eating Disorders | rs10954374 | A | G | 0.91728 | -0.11352 | 0.024832 | 4.84E-06 | 72,517 |
| Female Infertility | Eating Disorders | rs11056435 | A | G | 0.197031 | 0.092216 | 0.017396 | 1.15E-07 | 72,517 |
| Female Infertility | Eating Disorders | rs115338272 | C | T | 0.039841 | 0.159983 | 0.034637 | 3.86E-06 | 72,517 |
| Female Infertility | Eating Disorders | rs12806135 | G | A | 0.061973 | -0.14442 | 0.030178 | 1.7E-06 | 72,517 |
| Female Infertility | Eating Disorders | rs13028479 | T | G | 0.594588 | 0.067009 | 0.014407 | 3.3E-06 | 72,517 |
| Female Infertility | Eating Disorders | rs1424035 | A | C | 0.005481 | 0.457065 | 0.085265 | 8.3E-08 | 72,517 |
| Female Infertility | Eating Disorders | rs190946682 | A | G | 0.062927 | 0.198704 | 0.027811 | 9.02E-13 | 72,517 |
| Female Infertility | Eating Disorders | rs236332 | C | T | 0.246088 | 0.078294 | 0.016139 | 1.23E-06 | 72,517 |
| Female Infertility | Eating Disorders | rs2779747 | T | G | 0.577866 | -0.06692 | 0.014177 | 2.35E-06 | 72,517 |
| Female Infertility | Eating Disorders | rs313980 | A | C | 0.80806 | -0.0812 | 0.017652 | 4.22E-06 | 72,517 |
| Female Infertility | Eating Disorders | rs584336 | A | G | 0.299812 | 0.074614 | 0.015235 | 9.7E-07 | 72,517 |
| Female Infertility | Eating Disorders | rs6025 | T | C | 0.020732 | -0.24048 | 0.052434 | 4.51E-06 | 72,517 |
| Female Infertility | Eating Disorders | rs73527055 | C | T | 0.443192 | -0.0683 | 0.014213 | 1.55E-06 | 72,517 |
| Female Infertility | Eating Disorders | rs77893563 | A | G | 0.023406 | -0.22743 | 0.049285 | 3.94E-06 | 72,517 |
| Female Infertility | Eating Disorders | rs78721914 | G | A | 0.058209 | 0.134988 | 0.028992 | 3.22E-06 | 72,517 |

**Table S4. The Cochran's Q test for exposure on outcome**

| exposure | Outcome | MR‐Egger *p*-value | IVW *p*-value |
| --- | --- | --- | --- |
| Depression | Female infertility | 0.20 | 0.15 |
| Anxiety | Female infertility | 0.07 | 0.02 |
| Bipolar Disorder | Female infertility | 0.39 | 0.30 |
| Eating Disorders | Female infertility | 0.26 | 0.43 |
| Female infertility | Depression | 0.23 | 0.21 |
| Female infertility | Anxiety | 0.48 | 0.53 |
| Female infertility | Bipolar Disorder | 1.00 | 1.00 |
| Female infertility | Eating Disorders | 0.64 | 0.70 |

**Table S5. The MR-PRESSO test for** **exposure on outcome**

| exposure | Outcome | Global Test *p*-value |
| --- | --- | --- |
| Depression | Female infertility | 0.15 |
| Anxiety | Female infertility | 0.07 |
| Bipolar Disorder | Female infertility | 0.41 |
| Eating Disorders | Female infertility | - |
| Female infertility | Depression | 0.24 |
| Female infertility | Anxiety | 0.54 |
| Female infertility | Bipolar Disorder | 1.00 |
| Female infertility | Eating Disorders | 0.71 |

**Table S6. The MR-Egger intercept test for exposure on outcome**

| exposure | Outcome | egger-intercept | se | *p*-value |
| --- | --- | --- | --- | --- |
| Depression | Female infertility | 0.025 | 0.015 | 0.11 |
| Anxiety | Female infertility | 0.041 | 0.029 | 0.26 |
| Bipolar Disorder | Female infertility | 0.047 | 0.033 | 0.18 |
| Eating Disorders | Female infertility | 0.040 | 0.072 | 0.68 |
| Female infertility | Depression | 0.003 | 0.003 | 0.23 |
| Female infertility | Anxiety | -0.019 | 0.038 | 0.63 |
| Female infertility | Bipolar Disorder | 0.004 | 0.008 | 0.62 |
| Female infertility | Eating Disorders | 0.003 | 0.011 | 0.76 |
