# Supplementary material for: The causal relationship of female infertility and psychiatric disorders in the European population: a bidirectional two-sample Mendelian randomization study
Source: BMC Womens Health. 2024 Jan 19;24:54. doi: 10.1186/s12905-024-02888-5 (PMC10797979; doi:10.1186/s12905-024-02888-5)
Supplement: Supplementary file 2 — Additional file 2: Figure S1. MR analysis for depression on female infertility. (a) Scatter plots from depression on female infertility (b) Funnel plot from depression on female infertility (c) Forest plot from depression on female infertility (d) Leave-one-out plot from depression on female infertility. Figure S2. MR analysis for anxiety on female infertility (a) Scatter plots from anxiety on female infertility (b) Funnel plot from anxiety on female infertility; (c) Forest plot from anxiety on female infertility (d) Leave-one-out plot from anxiety on female infertility. Figure S3. MR analysis for bipolar disorder on female infertility (a) Scatter plots from bipolar disorder on female infertility; (b) Funnel plot from bipolar disorder on female infertility (c) Forest plot from bipolar disorder on female infertility (d) Leave-one-out plot from bipolar disorder on female infertility. Figure S4. MR analysis for eating disorders on female infertility (a) Scatter plots from eating disorders on female infertility; (b) Funnel plot from eating disorders on female infertility. (c) Forest plot from eating disorders on female infertility (d) Leave-one-out plot from eating disorders on female infertility. Figure S5. MR analysis for female infertility on depression (a) Scatter plots from female infertility on depression; (b) Funnel plot from female infertility on depression (c) Forest plot from female infertility on depression (d) Leave-one-out plot from female infertility on depression. Figure S6. MR analysis for female infertility on anxiety (a) Scatter plots from female infertility on anxiety (b) Funnel plot from female infertility on anxiety; (c) Forest plot from female infertility on anxiety (d) Leave-one-out plot from female infertility on anxiety. Figure S7. MR analysis for female infertility on bipolar disorder (a) Scatter plots from female infertility on bipolar disorder; (b) Funnel plot from female infertility on bipolar disorder; (c) Forest plot from female infertilit [file 12905_2024_2888_MOESM2_ESM.docx]

| 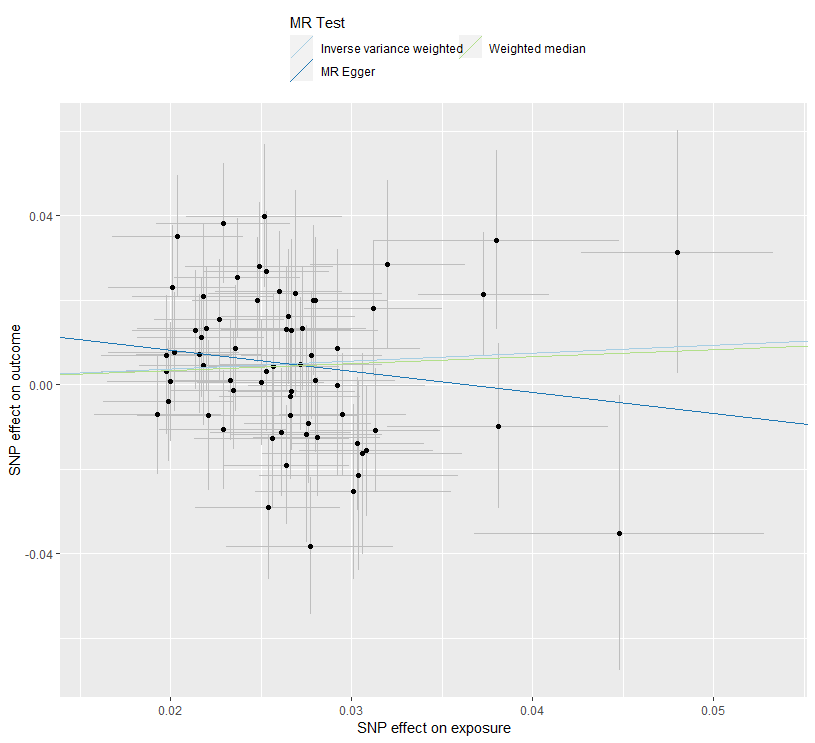 | 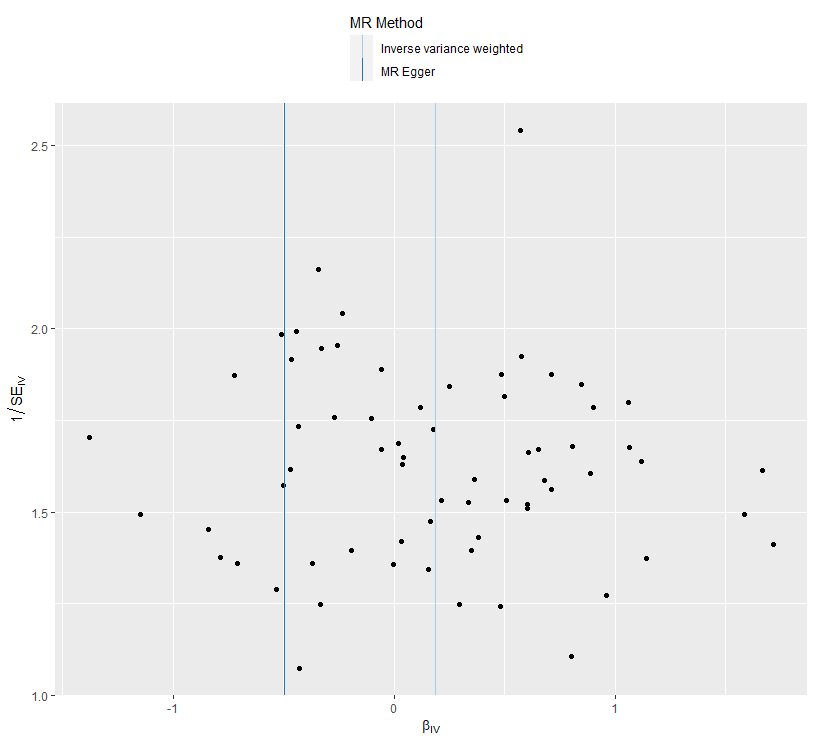 |
| --- | --- |
| A | B |
| 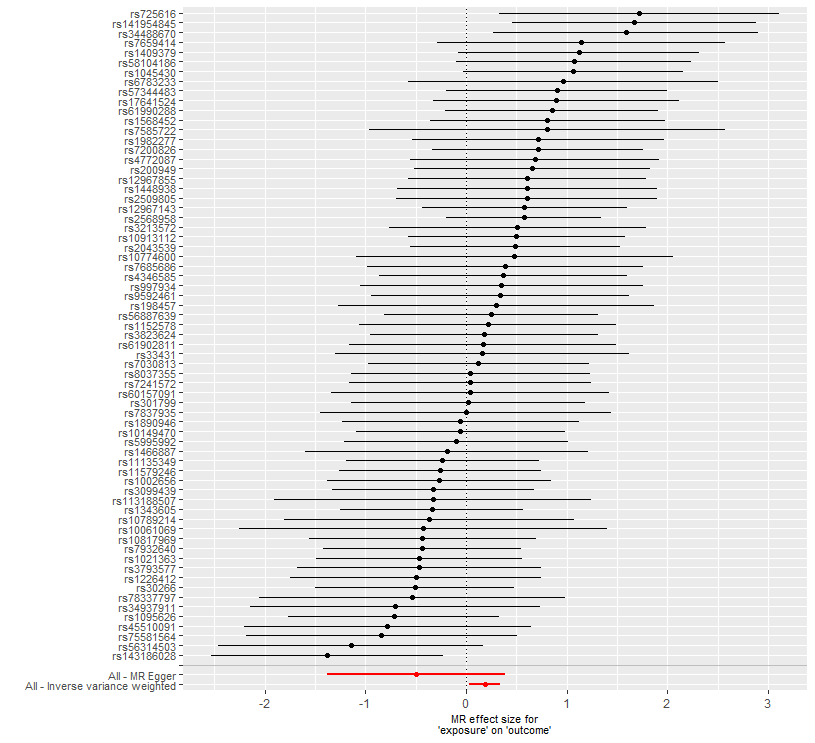 | 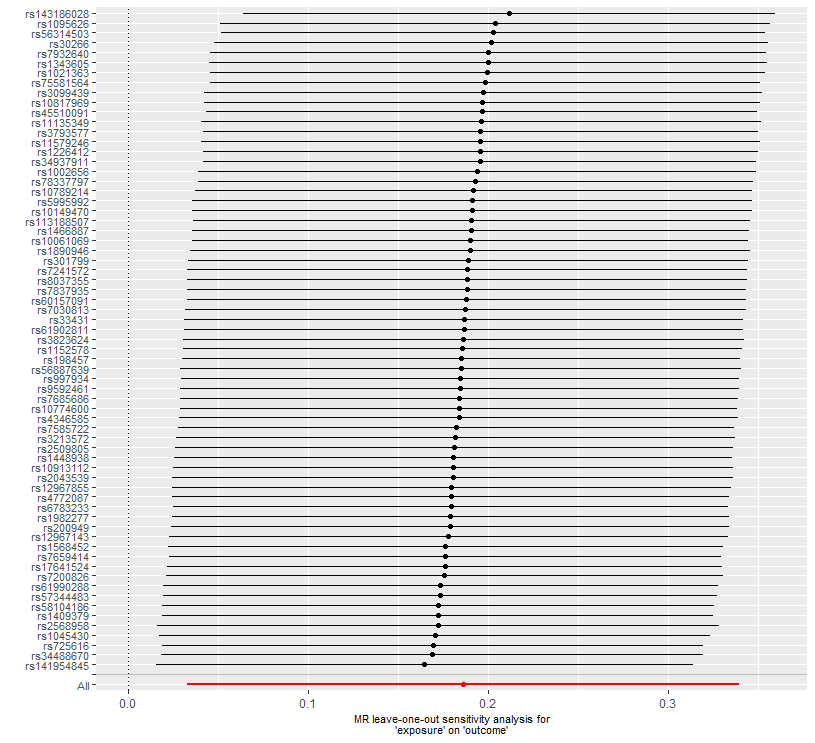 |
| C | D |

Figure S1 MR analysis for depression on female infertility. (A) Scatter plots from depression on female infertility; (B) Funnel plot from depression on female infertility; (C) Forest plot from depression on female infertility;(D) Leave-one-out plot from depression on female infertility.

| 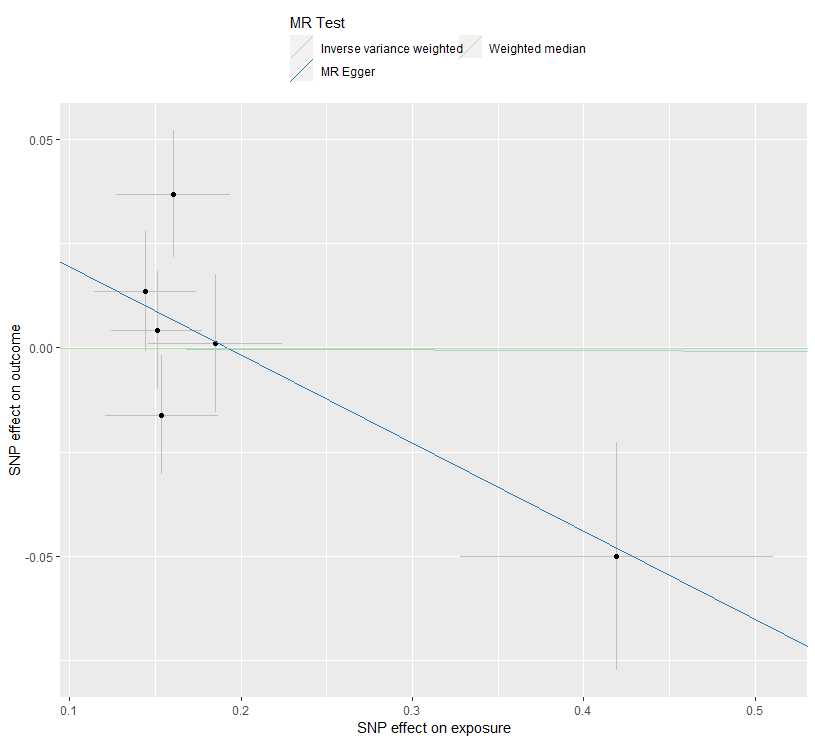 | 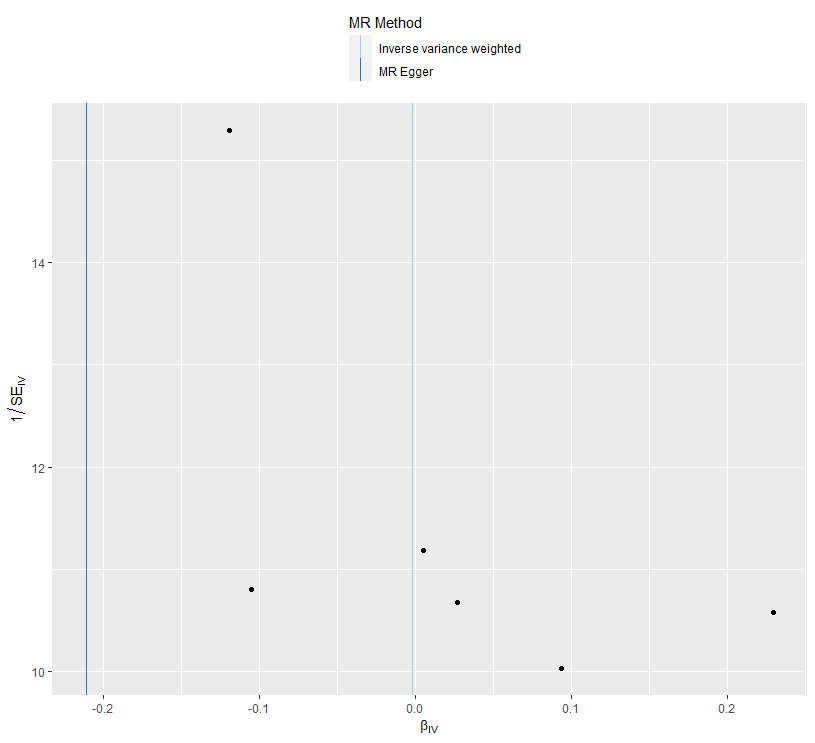 |
| --- | --- |
| A | B |
| 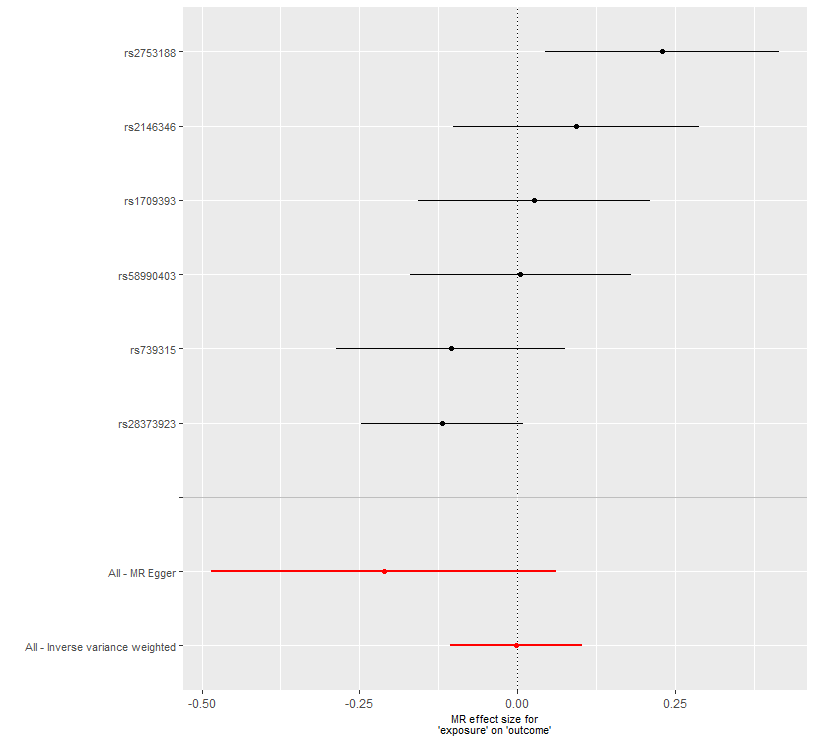 | 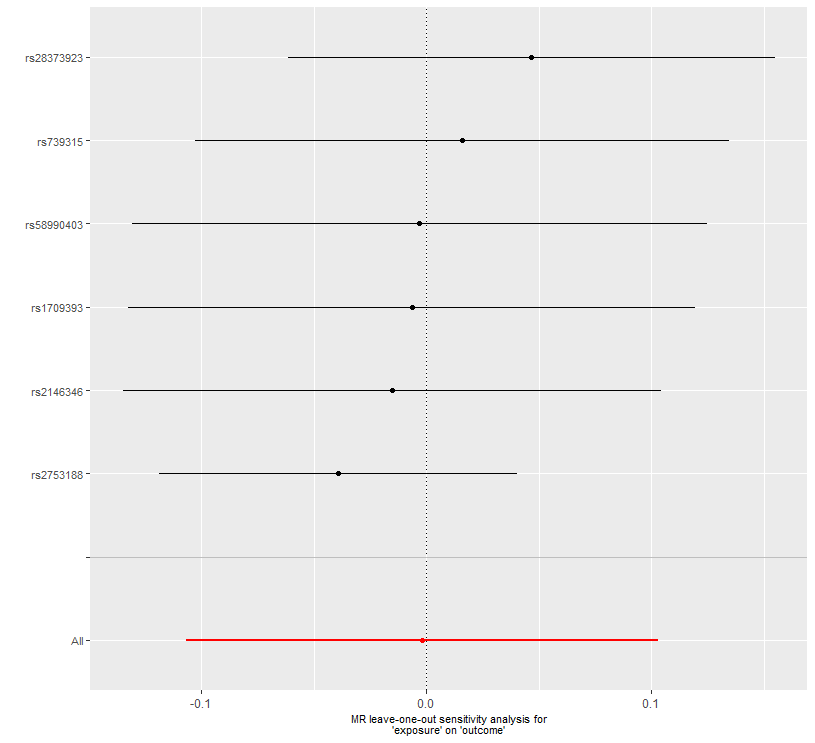 |
| C | D |

Figure S2 MR analysis for anxiety on female infertility. (A) Scatter plots from anxiety on female infertility; (B) Funnel plot from anxiety on female infertility; (C) Forest plot from anxiety on female infertility;(D) Leave-one-out plot from anxiety on female infertility.

| 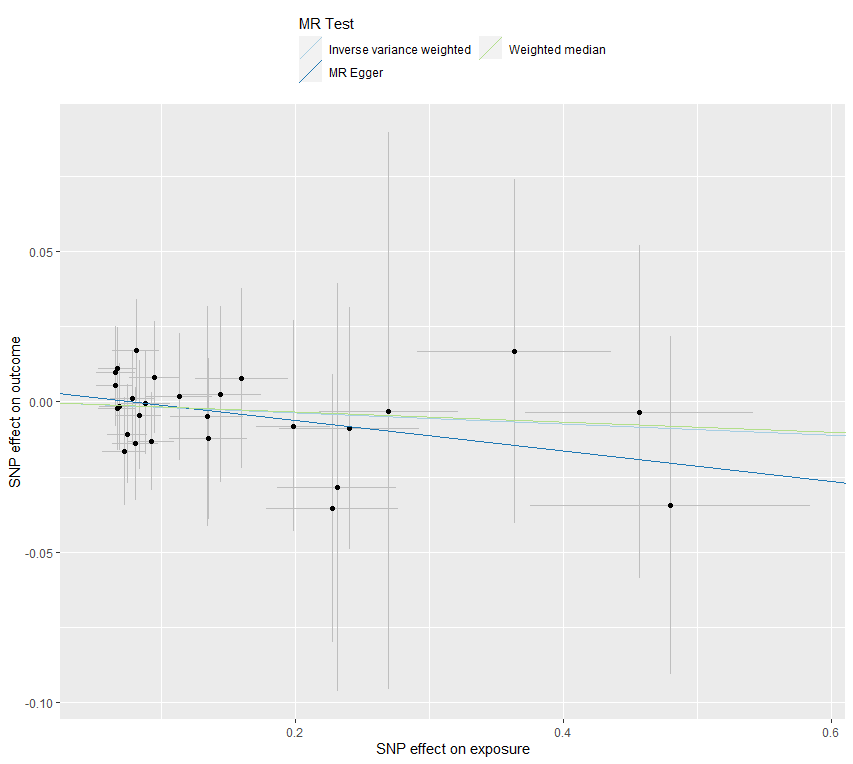 | 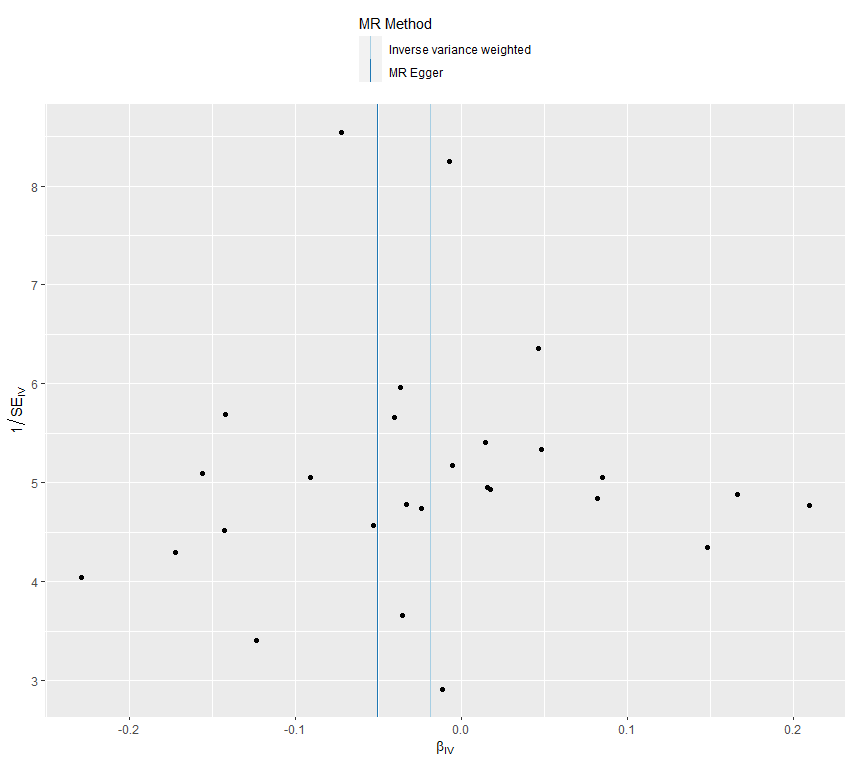 |
| --- | --- |
| A | B |
| 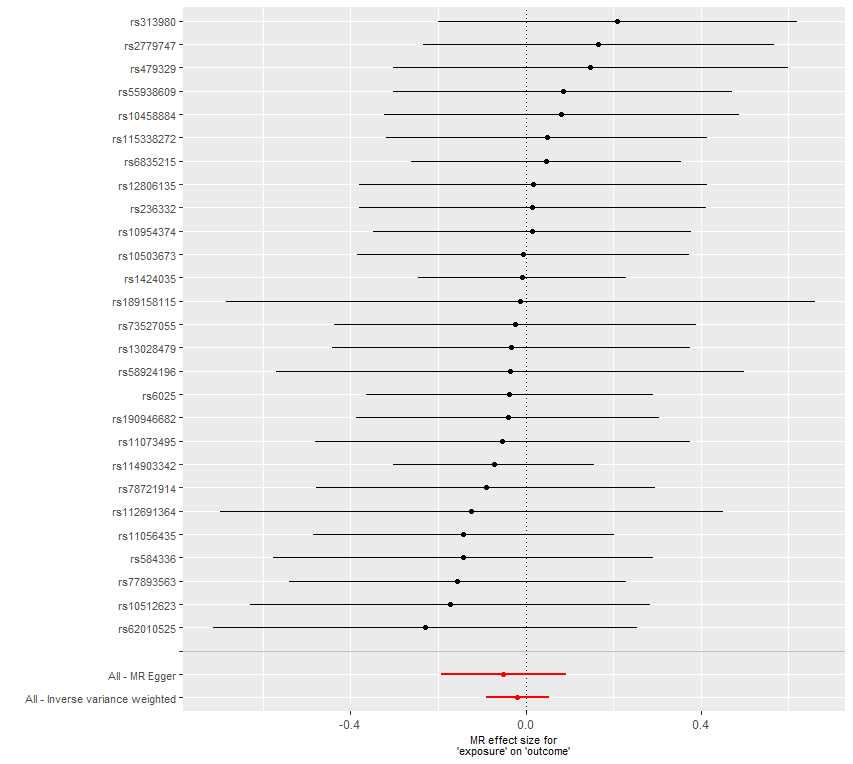 | 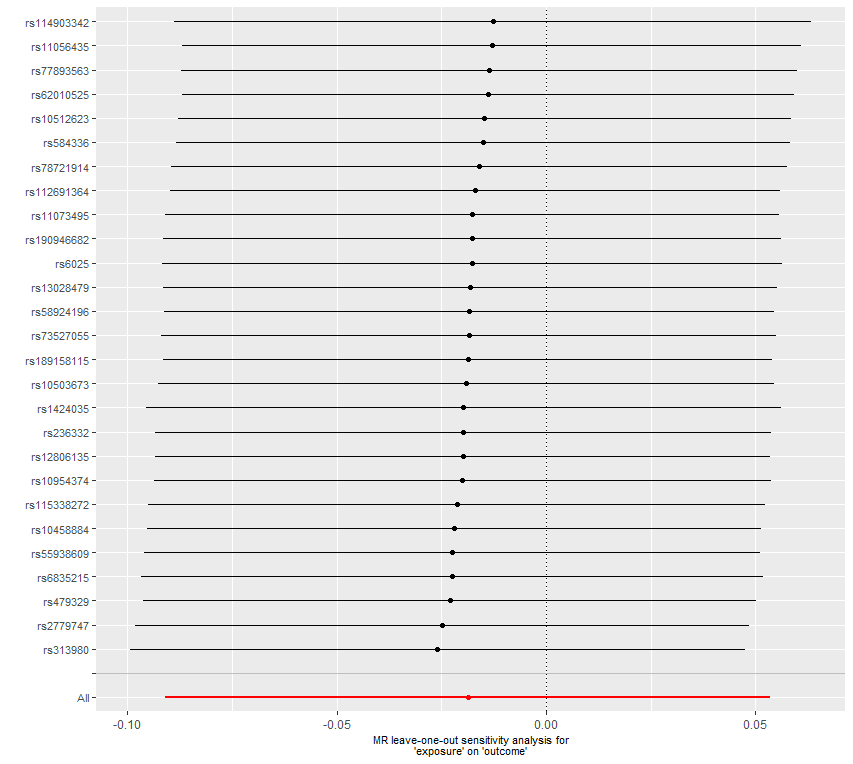 |
| C | D |

Figure S3 MR analysis for bipolar disorder on female infertility. (A) Scatter plots from bipolar disorder on female infertility; (B) Funnel plot from bipolar disorder on female infertility; (C) Forest plot from bipolar disorder on female infertility;(D) Leave-one-out plot from bipolar disorder on female infertility.

| 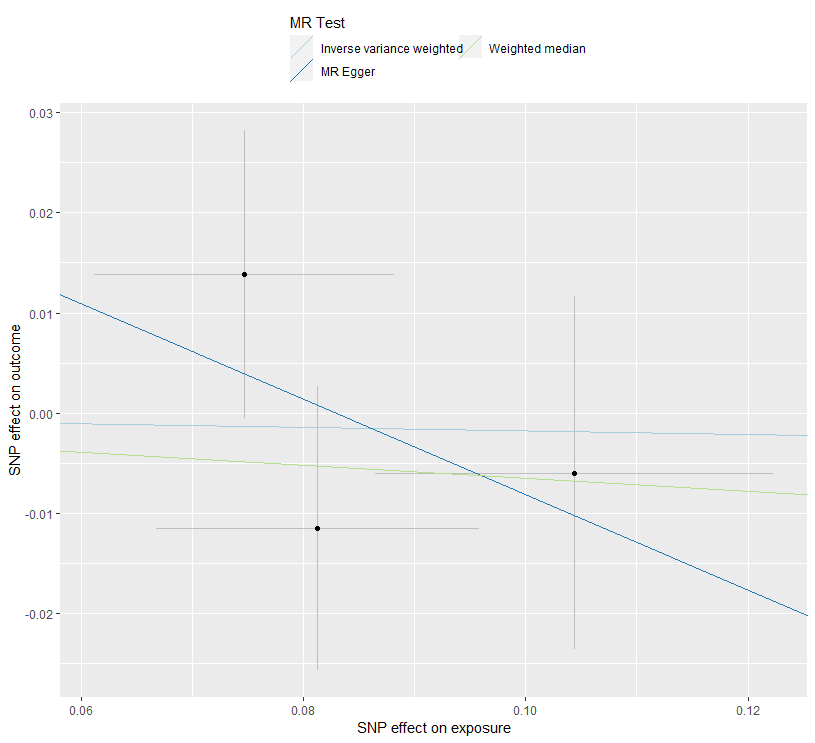 | 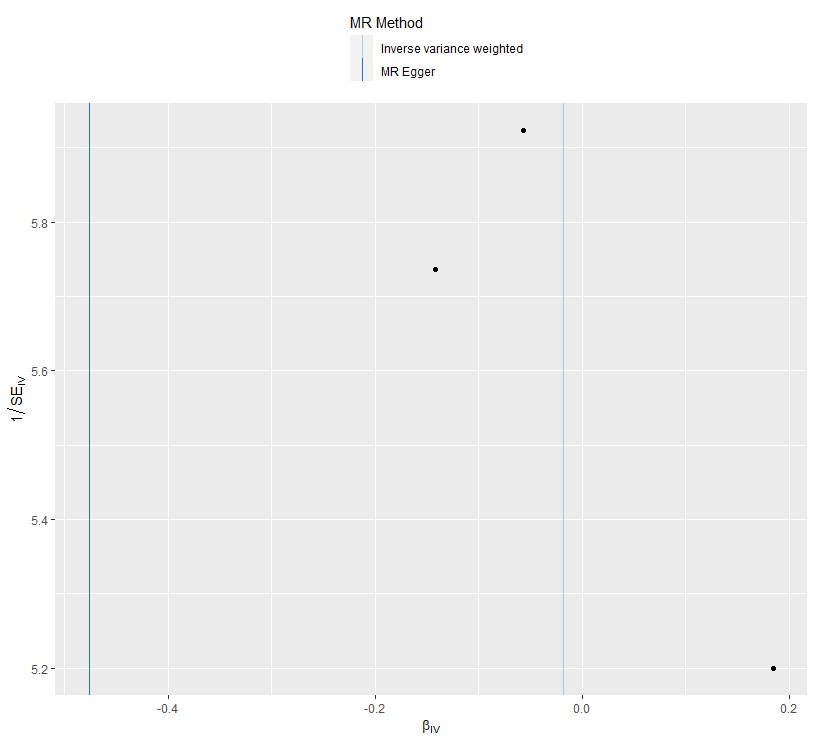 |
| --- | --- |
| A | B |
| 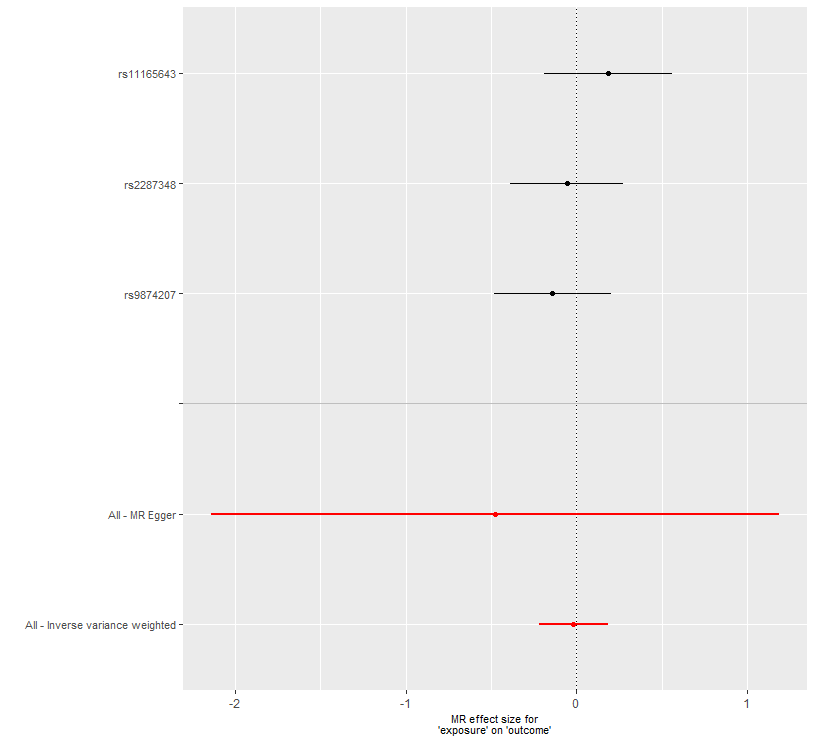 | 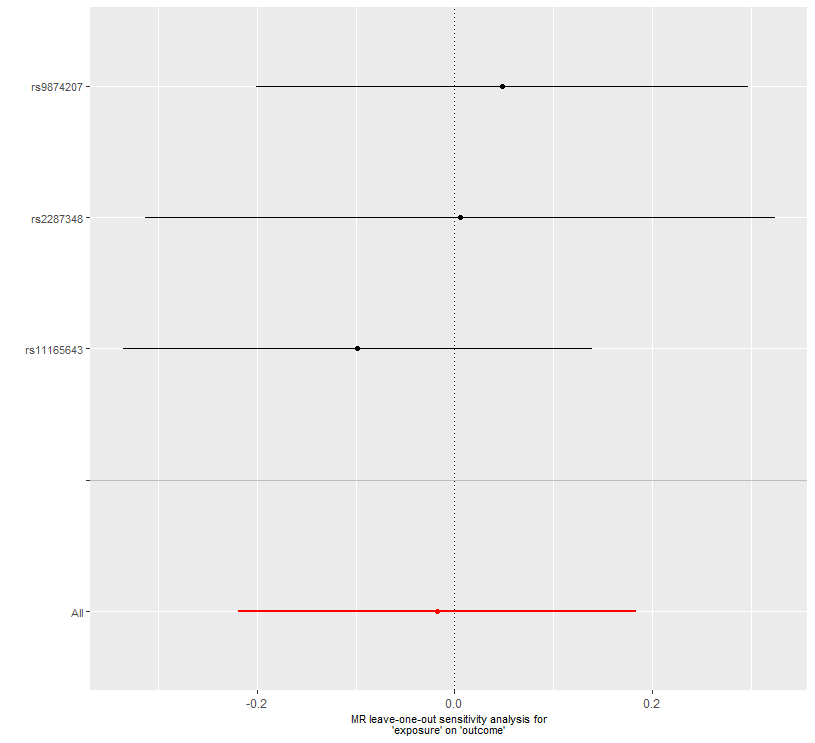 |
| C | D |

Figure S4 MR analysis for eating disorders on female infertility. (A) Scatter plots from eating disorders on female infertility; (B) Funnel plot from eating disorders on female infertility; (C) Forest plot from eating disorders on female infertility;(D) Leave-one-out plot from eating disorders on female infertility.

| 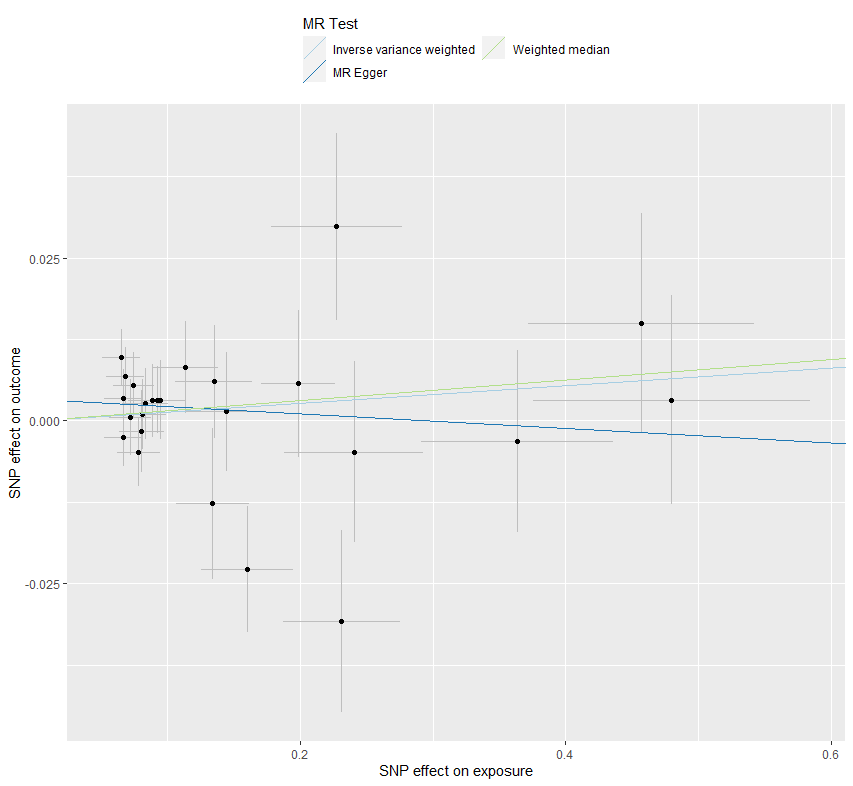 | 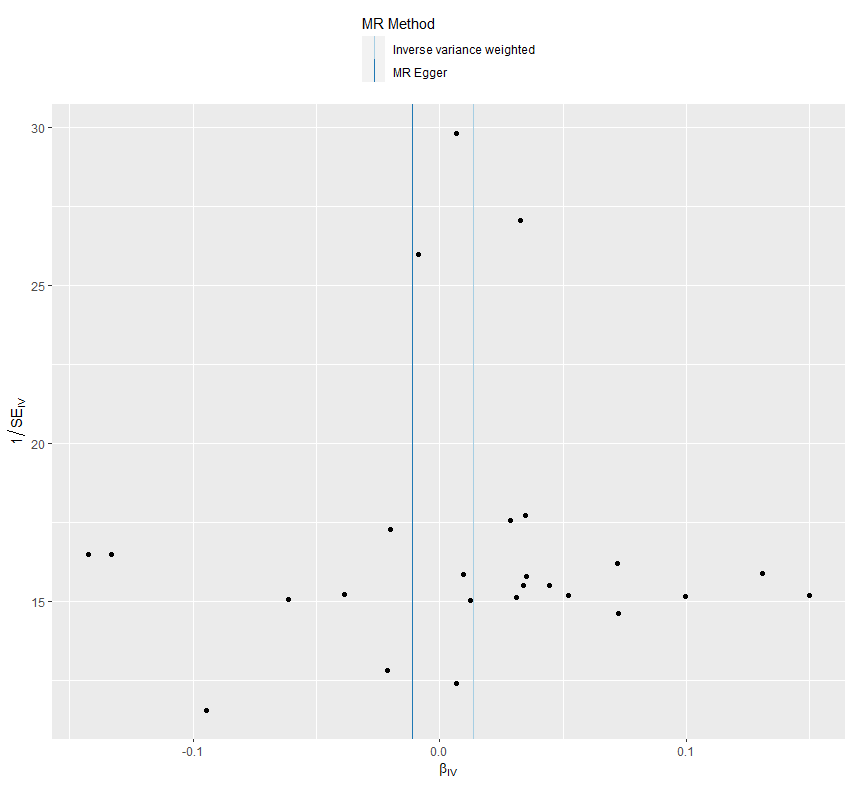 |
| --- | --- |
| A | B |
| 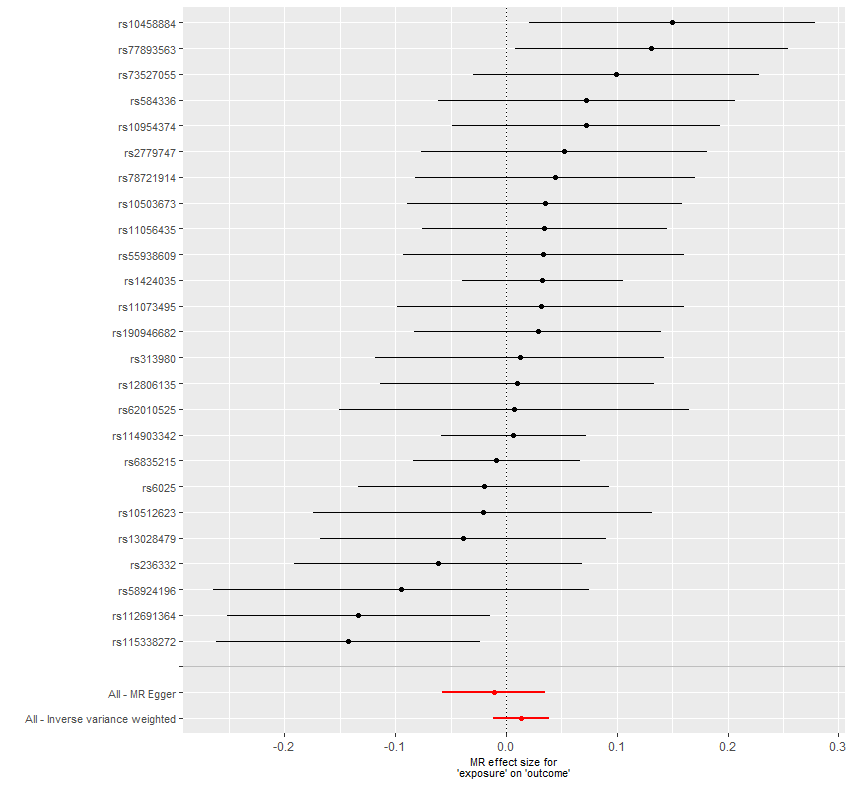 | 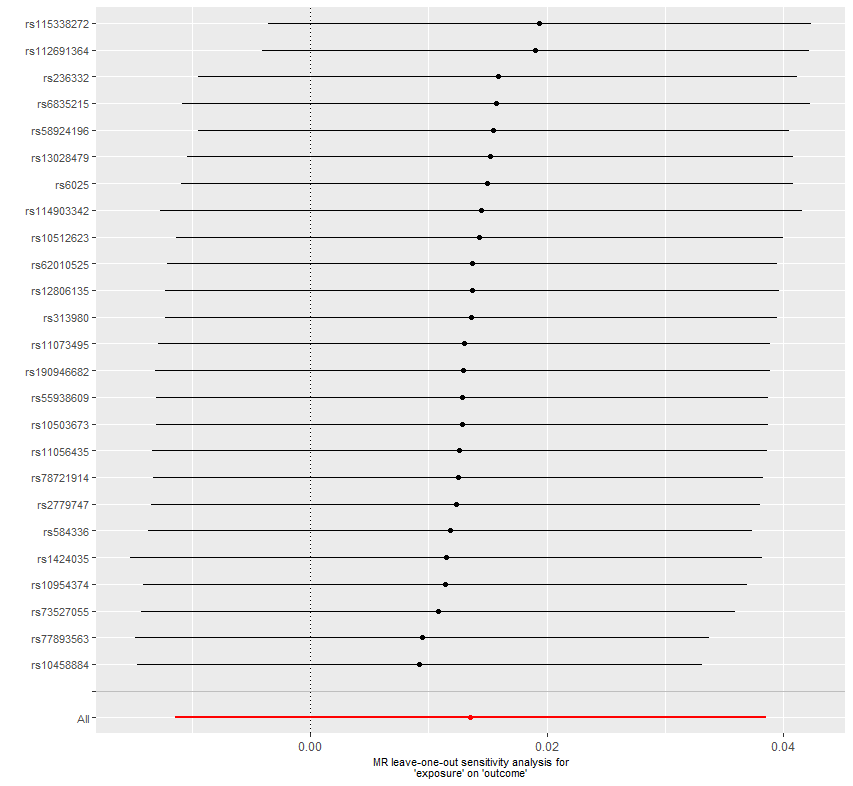 |
| C | D |

Figure S5 MR analysis for female infertility on depression. (A) Scatter plots from female infertility on depression; (B) Funnel plot from female infertility on depression; (C) Forest plot from female infertility on depression;(D) Leave-one-out plot from female infertility on depression.

| 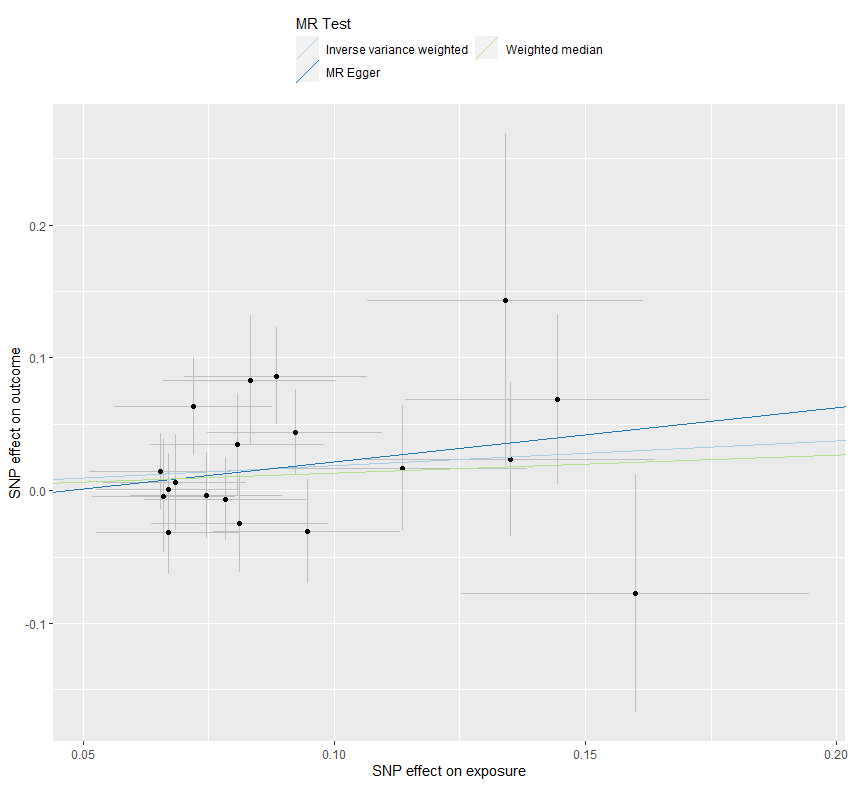 | 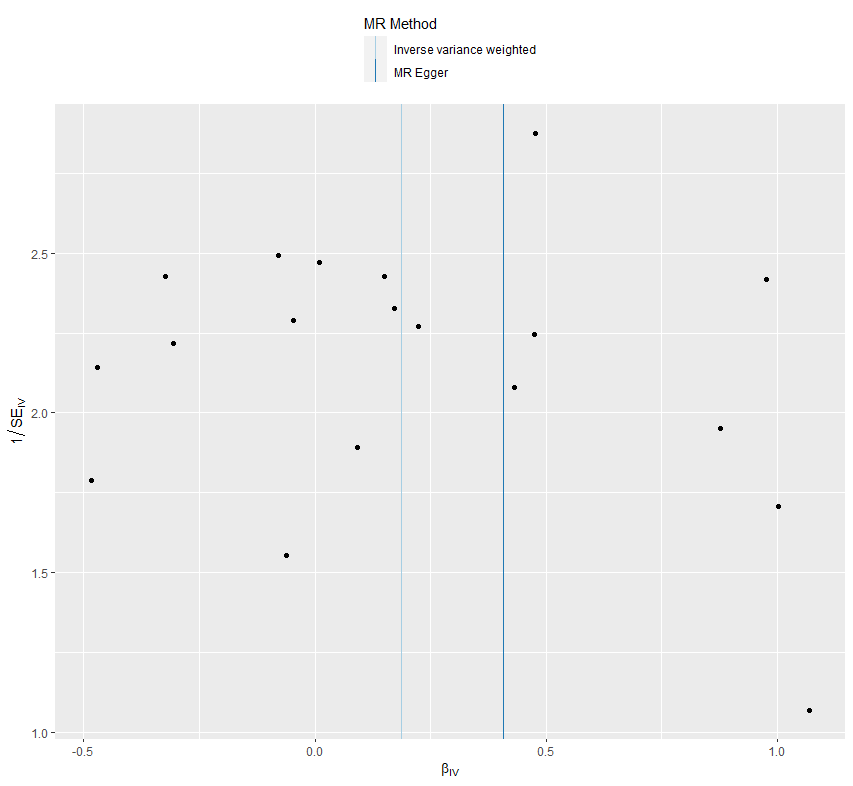 |
| --- | --- |
| A | B |
| 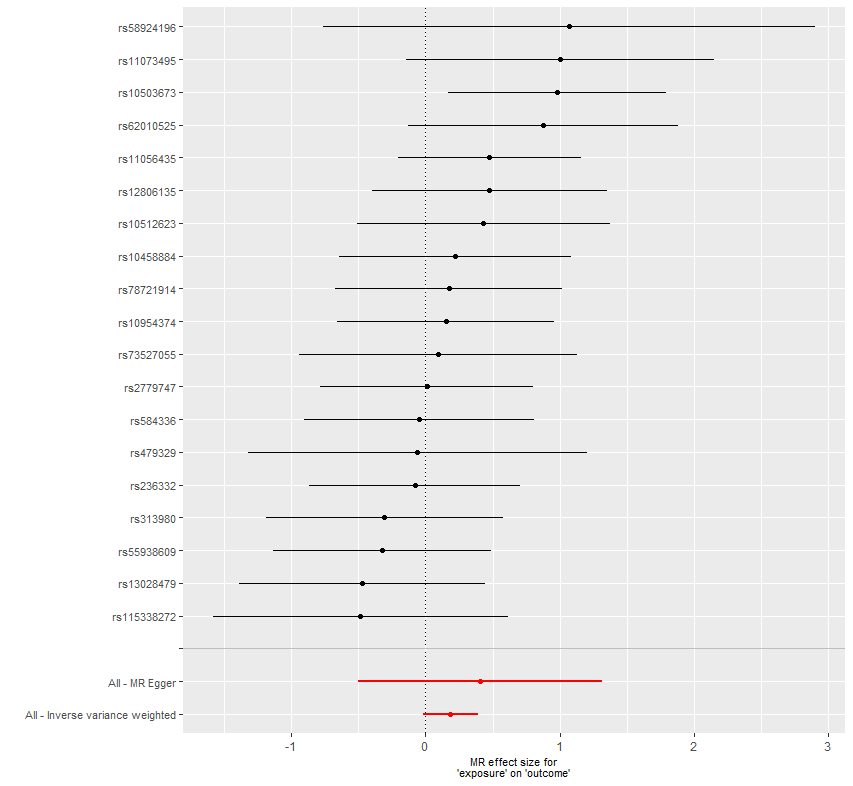 | 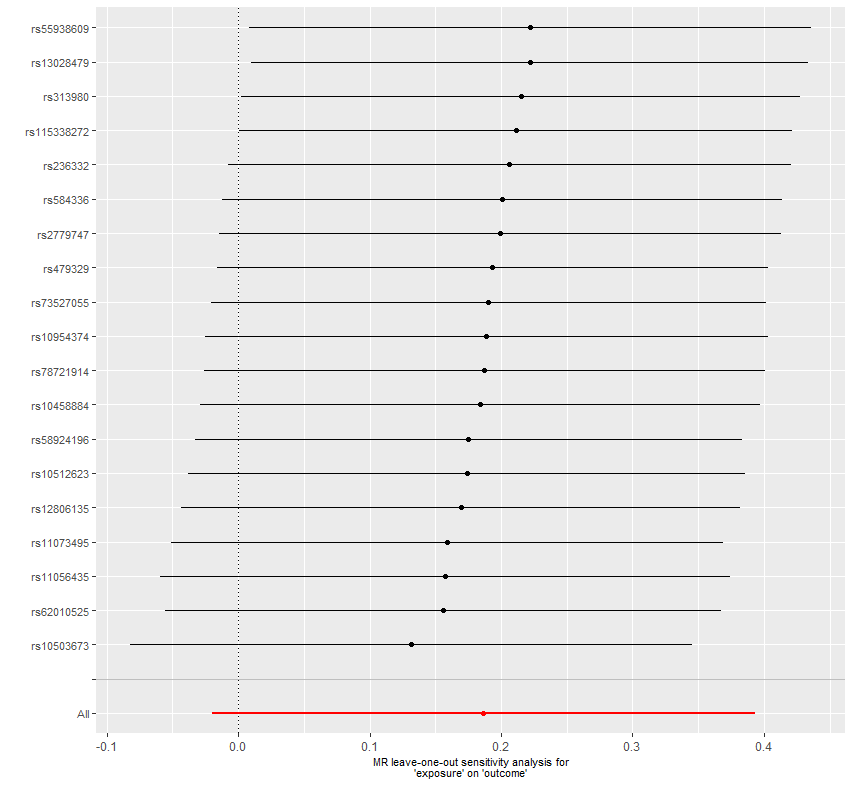 |
| C | D |

Figure S6 MR analysis for female infertility on anxiety. (A) Scatter plots from female infertility on anxiety; (B) Funnel plot from female infertility on anxiety; (C) Forest plot from female infertility on anxiety;(D) Leave-one-out plot from female infertility on anxiety.

| 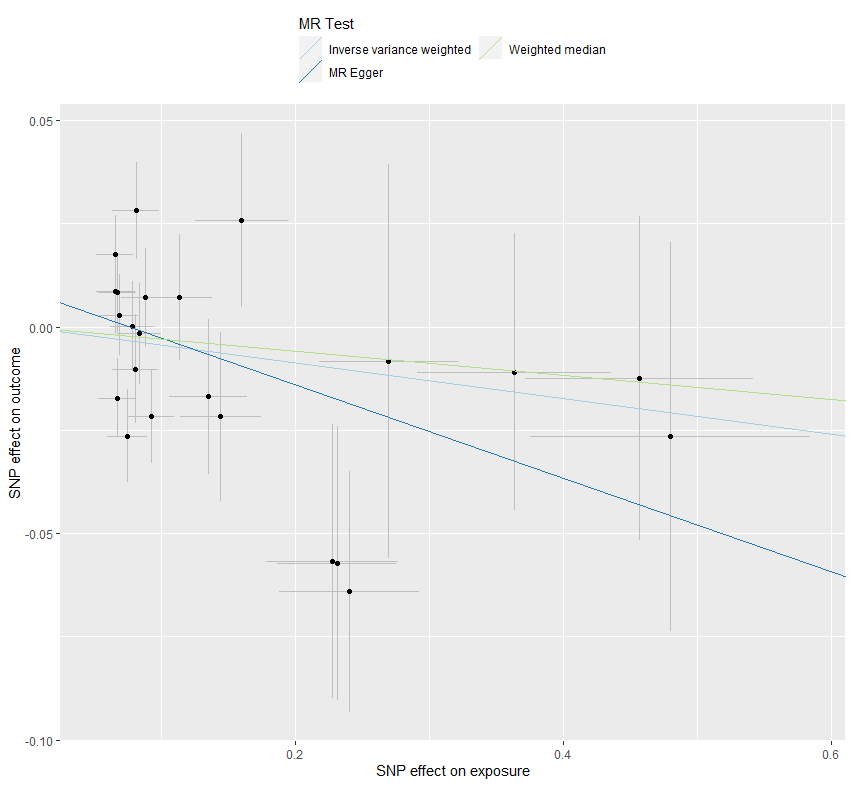 | 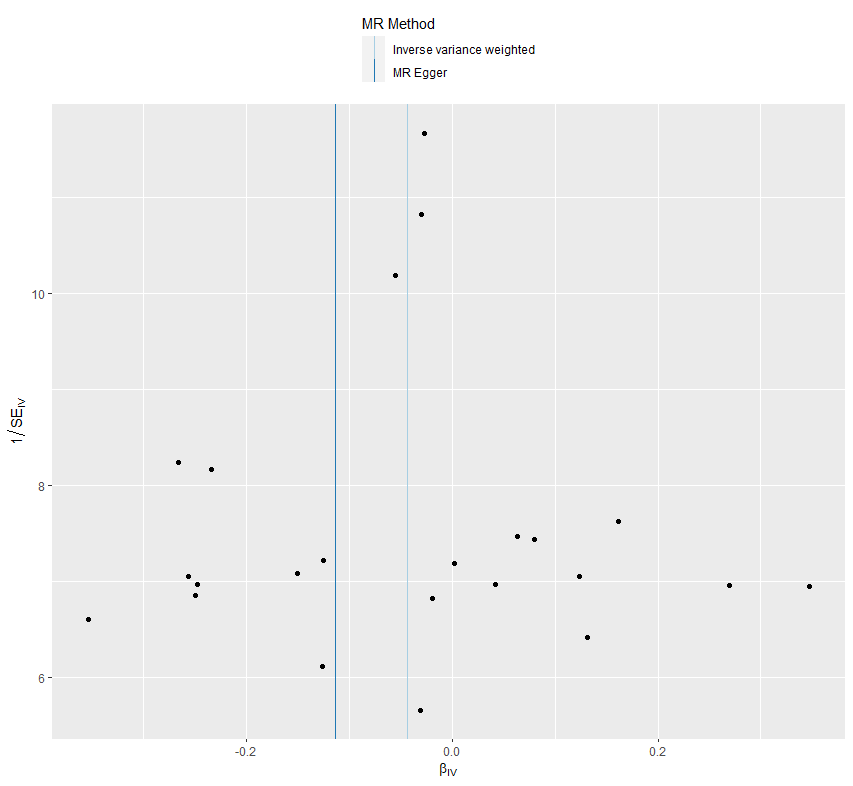 |
| --- | --- |
| A | B |
| 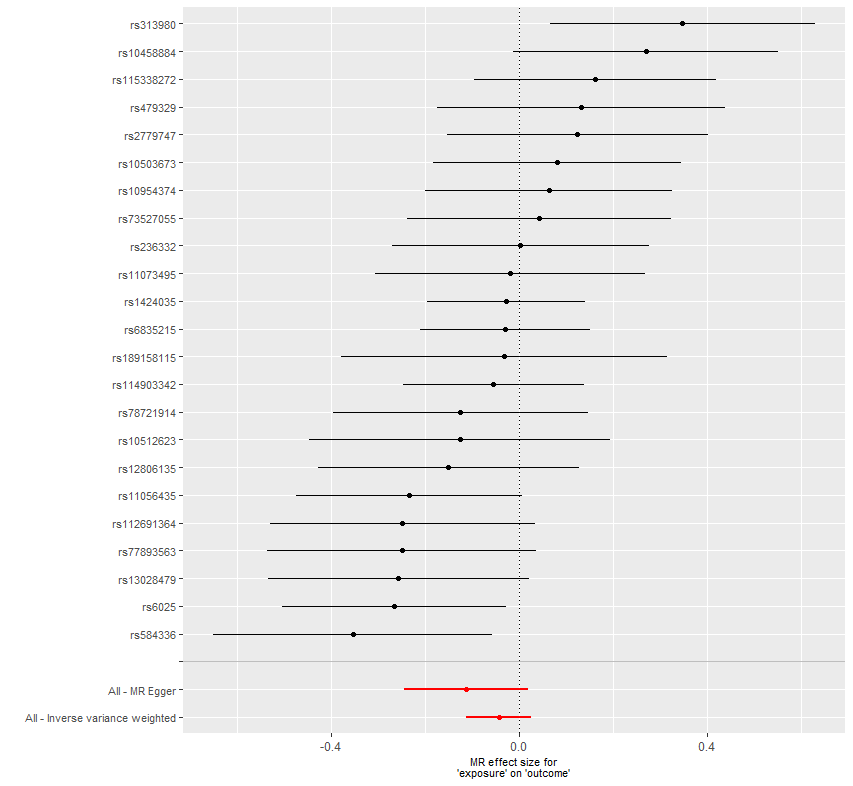 | 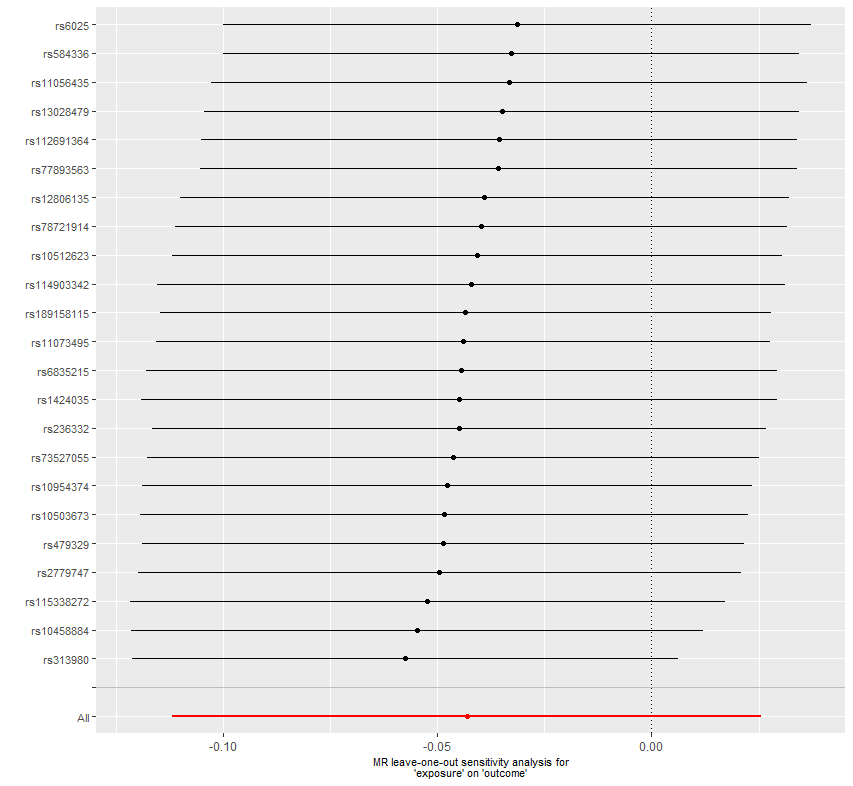 |
| C | D |

Figure S7 MR analysis for female infertility on bipolar disorder. (A) Scatter plots from female infertility on bipolar disorder; (B) Funnel plot from female infertility on bipolar disorder; (C) Forest plot from female infertility on bipolar disorder;(D) Leave-one-out plot from female infertility on bipolar disorder.

| 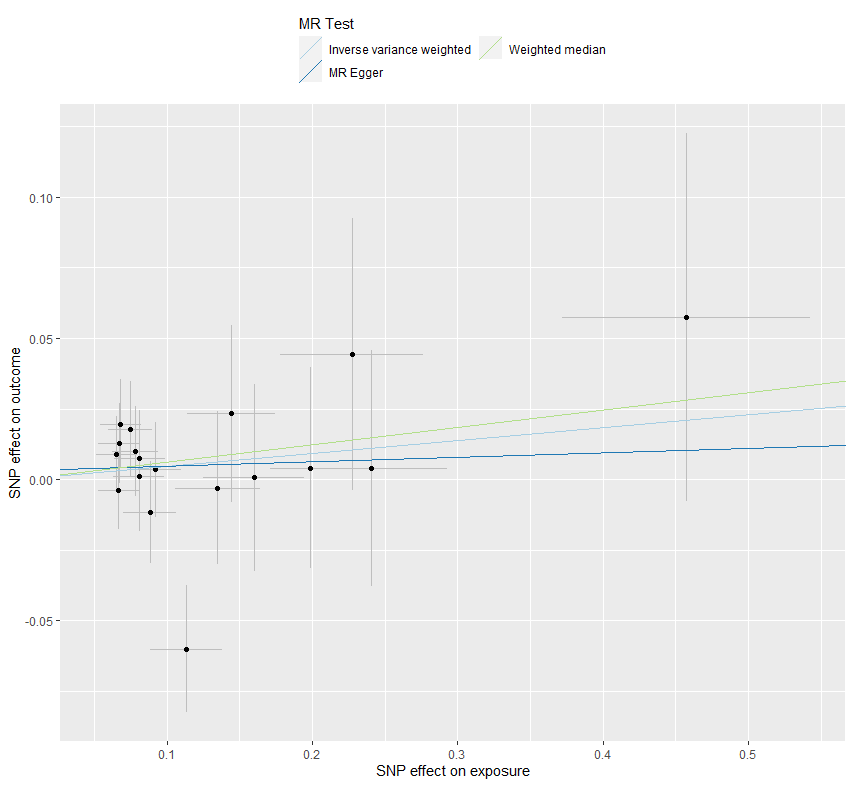 | 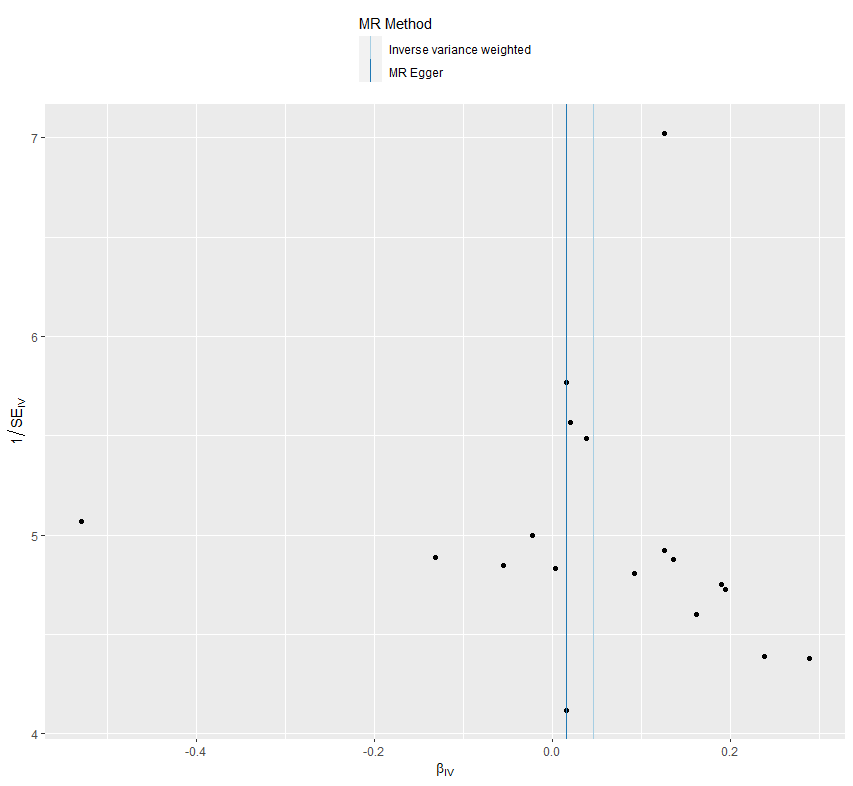 |
| --- | --- |
| A | B |
| 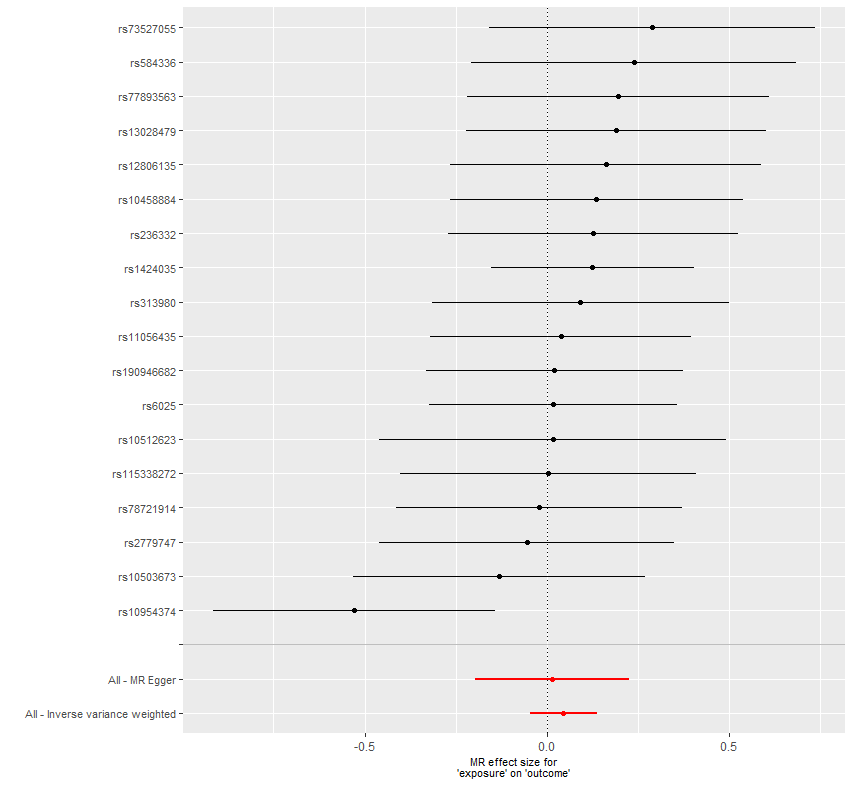 | 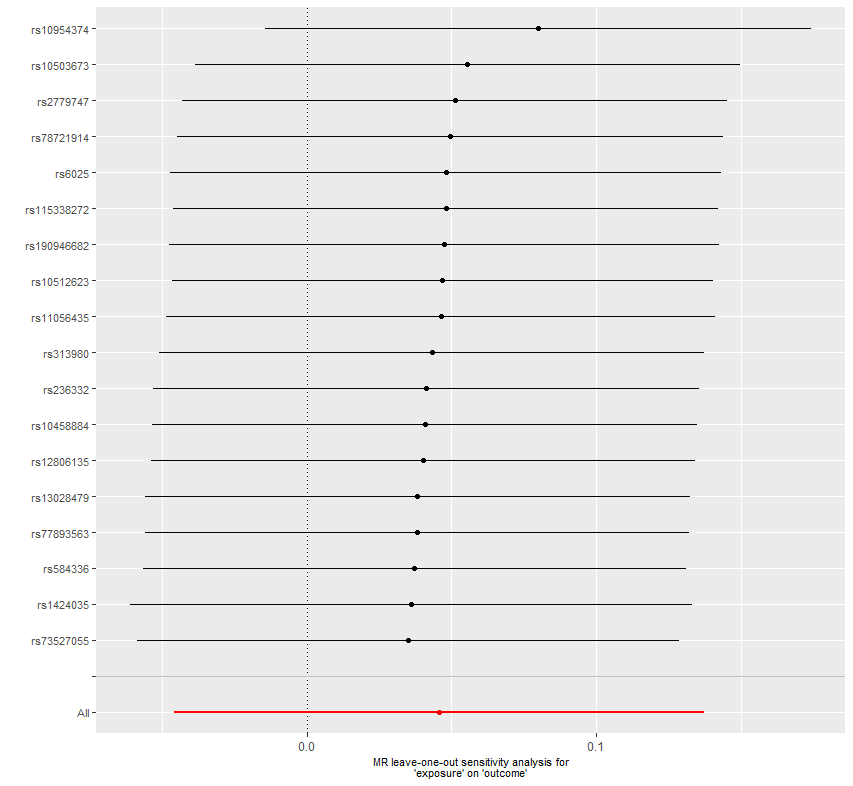 |
| C | D |

Figure S8 MR analysis for female infertility on eating disorders. (A) Scatter plots from female infertility on eating disorders; (B) Funnel plot from female infertility on eating disorders; (C) Forest plot from female infertility on eating disorders;(D) Leave-one-out plot from female infertility on eating disorders.
